# JC Polyomavirus Abundance and Distribution in Progressive Multifocal Leukoencephalopathy (PML) Brain Tissue Implicates Myelin Sheath in Intracerebral Dissemination of Infection

**DOI:** 10.1371/journal.pone.0155897

**Published:** 2016-05-18

**Authors:** Keith A. Wharton, Catherine Quigley, Marian Themeles, Robert W. Dunstan, Kathryn Doyle, Ellen Cahir-McFarland, Jing Wei, Alex Buko, Carl E. Reid, Chao Sun, Paul Carmillo, Gargi Sur, John P. Carulli, Keith G. Mansfield, Susan V. Westmoreland, Susan M. Staugaitis, Robert J. Fox, Werner Meier, Susan E. Goelz

**Affiliations:** 1 Translational Pathology Laboratory, Biogen Inc., Cambridge, MA, United States of America; 2 Immunology, Biogen Inc., Cambridge, MA, United States of America; 3 Bioanalytical Chemistry, Biogen Inc., Cambridge, MA, United States of America; 4 Molecular Discovery, Biogen Inc., Cambridge, MA, United States of America; 5 Department of Pathology, Harvard Medical School, New England Primate Research Center, Southborough, MA, United States of America; 6 Departments of Pathology, Neurosciences, and Mellen Center for Multiple Sclerosis, Cleveland Clinic, Cleveland, OH, United States of America; 7 Mellen Center for Multiple Sclerosis, Cleveland Clinic, Cleveland, OH, United States of America; 8 Discovery Sciences, Biogen Inc, Cambridge, MA, United States of America; 9 Neurology, Biogen Inc, Cambridge, MA, United States of America; National Institutes of Health, UNITED STATES

## Abstract

Over half of adults are seropositive for JC polyomavirus (JCV), but rare individuals develop progressive multifocal leukoencephalopathy (PML), a demyelinating JCV infection of the central nervous system. Previously, PML was primarily seen in immunosuppressed patients with AIDS or certain cancers, but it has recently emerged as a drug safety issue through its association with diverse immunomodulatory therapies. To better understand the relationship between the JCV life cycle and PML pathology, we studied autopsy brain tissue from a 70-year-old psoriasis patient on the integrin alpha-L inhibitor efalizumab following a ~2 month clinical course of PML. Sequence analysis of lesional brain tissue identified PML-associated viral mutations in regulatory (non-coding control region) DNA, capsid protein VP1, and the regulatory agnoprotein, as well as 9 novel mutations in capsid protein VP2, indicating rampant viral evolution. Nine samples, including three gross PML lesions and normal-appearing adjacent tissues, were characterized by histopathology and subject to quantitative genomic, proteomic, and molecular localization analyses. We observed a striking correlation between the spatial extent of demyelination, axonal destruction, and dispersion of JCV along white matter myelin sheath. Our observations in this case, as well as in a case of PML-like disease in an immunocompromised rhesus macaque, suggest that long-range spread of polyomavirus and axonal destruction in PML might involve extracellular association between virus and the white matter myelin sheath.

## Introduction

Progressive multifocal leukoencephalopathy (PML) is an opportunistic central nervous system (CNS) infection caused by JC polyomavirus (JCV) [1, 2]. Historically associated with immunosuppressive conditions such as AIDS and certain cancers, PML has more recently been linked to a variety of immunomodulatory therapies [3]. For example, Efalizumab (Raptiva^®^, Genentech/Merck Serono), a monoclonal antibody that targets the integrin α_L_ subunit CD11a/LFA1, blocks lymphocyte activation and egress from vasculature; it was approved in 2003 as therapy for moderate to severe plaque psoriasis but was withdrawn from the market in 2009 because of its association with PML and other infections [4]. Natalizumab (Tysabri^®^, Biogen), a monoclonal antibody against the integrin α_4_ subunit CD49d, is a highly effective therapy for multiple sclerosis and Crohn’s disease, but due to increased PML risk it is administered with diligent patient oversight [5]. Given the diversity of therapeutic strategies under development to manipulate the immune system and increasing numbers of patients using these therapies, PML and a growing list of polyomavirus-associated diseases will likely remain drug safety concerns for the foreseeable future.

JCV is non-enveloped virus with a 5.1 kB DNA genome that directs the synthesis of divergently transcribed early and late mRNAs to encode six proteins [6]. T-antigens (TAg) encoded by the early transcripts are expressed after JCV enters a target cell, co-opting cellular pathways necessary for viral DNA replication; late transcripts, which encode capsid proteins VP1, VP2, and VP3 as well as agnoprotein, are expressed after viral DNA replication and indicate a productive (virus-producing) infection [7]. JCV excreted in urine and spread through the human population retains an “archetype” (i.e., non-mutant) cis-regulatory noncoding control region (NCCR) that drives DNA replication and viral transcription in renal tubular and urothelial cells [8, 9]. JCV isolates from the brain or cerebrospinal fluid (CSF) of PML patients usually harbor unique NCCR mutations, deletions, and/or rearrangements, some of which have been shown to increase viral transcription in cultured cells of central nervous system origin; in addition, ~80–90% of PML JCV isolates carry VP1 point mutations [10–14], and mutations in agnoprotein have been identified in the PML-variant JCV encephalopathy [15]. Oligodendrocytes are the predominant cell type infected (and ultimately killed) by JCV in the majority of human PML patients, leading to extensive demyelination and tissue destruction, but depending on context other cell types including cerebellar granule neurons, cortical astrocytes, and even cortical neurons can exhibit productive infection [16–19]. How the immune system normally limits viral replication and spread of JCV into the CNS and how altered immunity allows JCV to spread throughout CNS white matter in PML are not known. To gain insight into PML pathogenesis, we studied brain tissues from a patient who died following a relatively rapid 2-month course of PML associated with chronic efalizumab therapy, and from an immunosuppressed rhesus macaque with SV40 polyomavirus-associated PML-like CNS disease.

## Materials and Methods

### Specimen characterization

**Fig 1:**
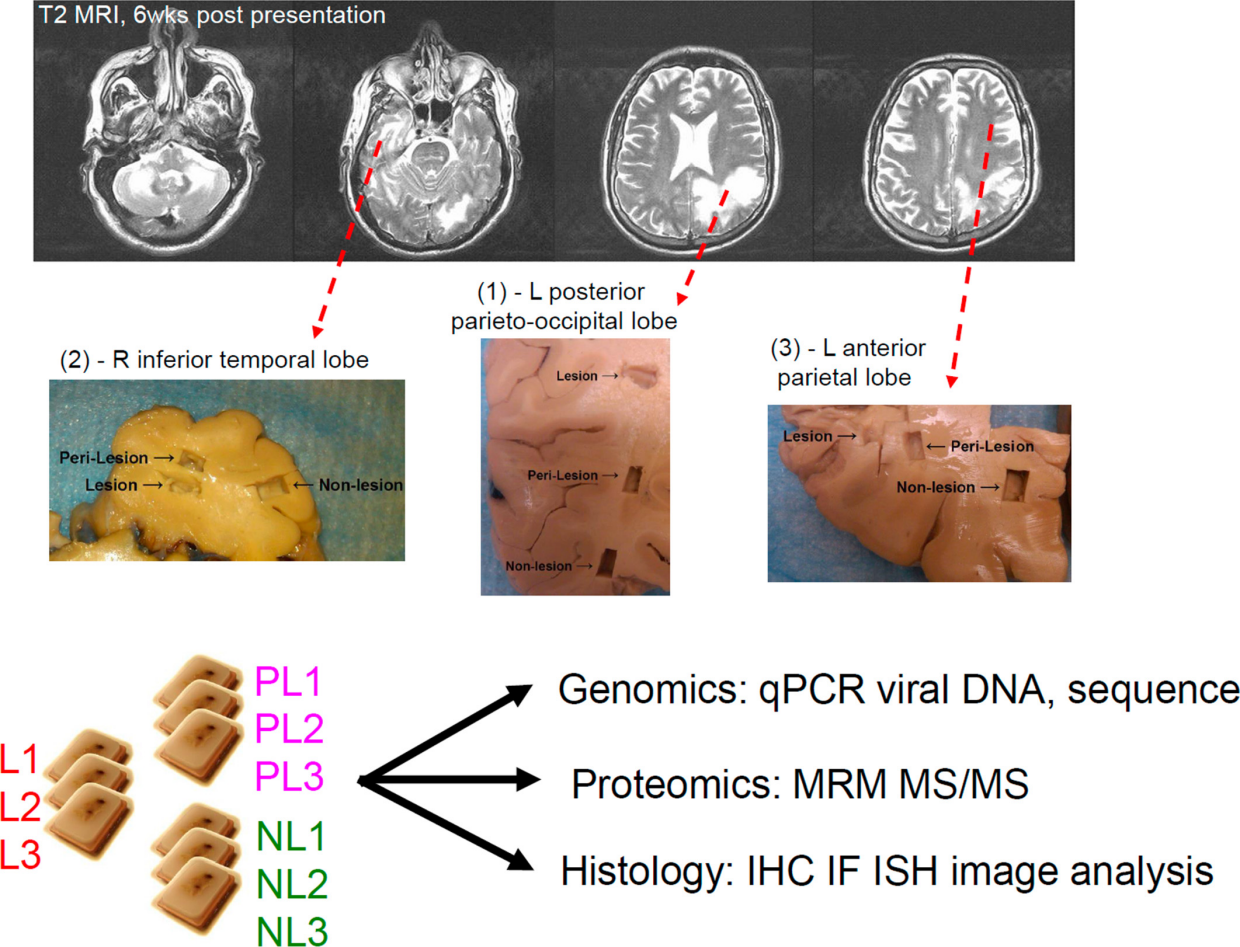
Tissue blocking and experimental scheme. Fixed postmortem sections corresponding to T2 brain MRI images at 6 weeks post-presentation, ~1.5 weeks prior to death. For indicated brain regions, L, PL, and NL tissue blocks, designated L1–3, PL1–3, and NL1–3, were studied as indicated. T2 - T2-weighted; qPCR—quantitative PCR; MRM/MS–multiple reaction monitoring mass spectrometry; IHC–immunohistochemistry; IF–immunofluorescence; ISH—in-situ hybridization.

Experiments on existing brain tissue samples from autopsy material consented for use in the research were part of an IRB-approved collaboration between Biogen and Cleveland Clinic Foundation (CCF IRB#: IRB 2076), in accordance with the principles expressed in the Declaration of Helsinki. Grossly lesional (L), perilesional (PL), and nonlesional (NL) formalin-fixed human PML brain tissue samples were excised from the patient’s 1) right inferior temporal lobe, 2) left posterior parieto-occipital lobe, and 3) left parietal lobe with fresh scalpels to minimize potential cross-contamination, processed, embedded in paraffin, and cut with fresh microtomy blades at 5-μm thickness for staining and at specified thicknesses for genomic and proteomic analyses; the sections were labeled L1–3, PL1–3, and NL1–3, respectively (Fig 1). Snap frozen lesional white matter collected at autopsy was processed for viral sequencing. Rhesus tissues from existing necropsy samples were from an 8-year-old female *Macaca mulatta* that developed neurologic signs 2 years post-Simian Immunodeficiency Virus (SIV) inoculation, in association with a National Institutes of Health-funded study approved by Institutional Animal Care and Use Committee of Harvard Medical School. Postmortem examination revealed PML-like disease of the CNS and multiorgan lymphoproliferative disease.

### Quantitative polymerase chain reaction (PCR)

DNA was extracted from 50-μm formalin-fixed paraffin-embedded (FFPE) tissue slices using the QIAamp DNA FFPE Tissue Kit according to the manufacturer’s protocol (#56404, Qiagen, Inc., Valencia, CA). Total DNA concentrations were measured by Picogreen assay (Invitrogen, Inc., Carlsbad, CA). JCV DNA was quantified in triplicate by Taqman quantitative PCR (qPCR) on a Stratagene MX3005P instrument (Agilent Technologies, Inc., Santa Clara, CA) using primers against TAg sequences. Negative control sections of multiple sclerosis and Alzheimer’s disease brain tissue in the qPCR assay had JCV DNA levels >100x lower than the PML block (NL3) with the lowest amount of virus (data not shown), and three non-PML brain sections showed no amplified JCV bands on gel electrophoresis (data not shown).

### JCV sequence analysis

Genomic DNA and total RNA were extracted from frozen lesional brain tissue using Qiagen AllPrep DNA/RNA Micro Kit (Cat 80284; Qiagen, Inc.). The Herculase II Fusion Enzyme system (#600677; Agilent Technologies, Inc.) was used to amplify overlapping VP1 (nucleotides 873–2899; 2026 base pairs) and NCCR-VP2 (nucleotides 5086–1722; 1766 base pairs) regions relative to JCV reference MAD1 archetype sequence NCBI NC_001699.1. The VP1 region was amplified with primers 5′-GCAGCCAGCTATGGCTTTAC-3′ and 5′-GCTGCCATTCATGAGAGGAT-3′ and NCCR-VP2 region was amplified with: 5′-CCTCCACGCCCTTACTACTTCTGAG-3′ and 5′-CTGGCCACACTGTAACAAGG-3’ then amplification products were cloned using the TOPO TA Cloning Kit (#K4575J10; Life Technologies). Ligation products were transformed into E. coli, plated on selective LB-agar, and each colony sequenced on an Applied Biosystems 3730XL using v3.1 chemistry. Representative amplified and aligned DNA sequences are shown in S1 File and have been deposited at NCBI under the accession nos.KX216358-KX216371.

### Proteomics

FFPE tissue slices (20 μm thick) were deparaffinized in xylenes/ethanol, and proteins extracted at 95°C × 20 min in 150 mM NaCl, 50 mM (4-(2-hydroxyethyl)-1-piperazineethanesulfonic acid (HEPES), 1 mM ethylenediaminetetraacetic acid (EDTA), 4% Rapigest (Waters Corp., Milford, MA), pH 7.4, and then digested at 80°C × 2 h in 150 mM NaCl, 50 mM HEPES, 1 mM EDTA, pH 7.4. Samples were sequentially deglycosylated (EMD Chemicals, Cambridge, MA), proteolyzed (Lys-C and trypsin, Roche Applied Science), and reduced in Tris(2-carboxyethyl)phosphine. Protein yields were determined before and after digestions using a Bio-Rad DC Protein Assay kit (Life Science, Hercules, CA). Liquid chromatography was performed on an Accela ultra-high-performance liquid chromatography system (Thermo Fisher Scientific, Waltham, MA) and multiple reaction monitoring (MRM) mass spectrometry on a TSQ Vantage quadrupole mass spectrometer (Thermo Fisher Scientific) with 30 min acquisition time. Peptide spectra were generated in triplicate and analyzed using Skyline freeware (University of Washington, Seattle, WA).

### Immunohistochemistry (IHC) / immunofluorescence (IF)

Immunostains were performed on Discovery XT (Ventana Medical Systems, Inc., Tuscon, AZ) or Dako (Glostrup, Denmark) automated platforms using borate (“CC1-standard”) or citrate buffer-based antigen retrievals, respectively. No autofluorescence quenching techniques were employed. Each primary antibody was incubated on tissue sections for 1 hour at 37°C. Antibodies used: glial fibrillary acidic protein (GFAP; mouse monoclonal clone 6F2, Dako Cytomation M-0761, 1:40,000), myelin basic protein (MBP; rabbit polyclonal, Millipore [Billerica, MA] AB980, 1:250), neurofilament (mouse monoclonal clone NN18, Millipore MAB5254, 1:6,400), ionized calcium-binding adaptor molecule 1 (IBA1; mouse monoclonal, Wako [Osaka, Japan] #019–19741, 1:4,000), VP1 (mouse monoclonal PAB597, 0.01 to 1 μg/mL; rabbit polyclonal, Abcam [Cambridge, MA] ab53977, 1:500 to 1:8000), and TAg (rabbit polyclonal v-300, Santa Cruz Biotechnology [Santa Cruz, CA] sc-20800, 1:50). For secondary IHC detection, OmniMap antimouse horseradish peroxidase (HRP) or OmniMap antirabbit HRP (Ventana Medical Systems) with 3',3'-diamino benzidine (DAB) was used with hematoxylin counterstain. For the TAg/VP1 double IHC, VP1 was detected with HRP/DAB, and TAg was detected with alkaline phosphatase-conjugated secondary antibody and fast red substrate. For fluorescence detection, (alexa-fluor) AF488- or AF594-conjugated goat antirabbit or antimouse secondary antibodies (Invitrogen, A11001, A11005, A11008, A11012) were used at 1:200. 4',6-diamidino-2-phenylindole (DAPI) was used as a DNA counterstain. All control (non-PML) human brain tissues including those from patients with multiple sclerosis or Alzheimer’s disease showed no specific VP1 or TAg staining (example in S1 Fig).

### RNAscope in situ hybridization

Separate probes were designed to detect JCV early and late regions, and RNAscope ± RNAse treatment to detect viral DNA was performed by Advanced Cell Diagnostics, Inc. (Hayward, CA) as described [20], with DAB-based detection and hematoxylin counterstain. Both JCV probes generated similar staining patterns on each PML block, and neither probe detected BK virus, the most closely related human polyomavirus to JCV, in a case of BKV-associated polyomavirus nephropathy (data not shown). The negative control probe was dapB, a bacterial gene.

### Microscopy

Slides were scanned on a ScanScope (Aperio, Vista, CA) brightfield slide scanner. Individual slides were viewed on an Olympus BX53 microscope (Olympus Microscopy, Essex, UK) and images acquired on a Spot Insight 4.0-megapixel color digital camera (Spot Imaging Solutions, Sterling Heights, MI). Confocal image stacks were acquired on an inverted Zeiss LSM 710 confocal microscope (Carl Zeiss Ltd, Hertfordshire, UK).

### Image analysis and calculations

Image analysis was performed on whole slide images using Visiomorph (Visiopharm, Denmark). Hematoxylin and eosin (H&E)-stained sections were used to determine tissue areas (mm^2^), and section thicknesses (μm) were then used to calculate tissue volume (μL). Cells per μL of tissue were determined by dividing cell equivalents calculated from total DNA yield by the volume of each tissue slice analyzed by PCR. (At the highest viral concentrations in our set of 9 sections, viral DNA was <2% of total DNA, allowing total DNA to be used as an estimate of cellular DNA; calculation not shown.) JCV capsid concentrations were calculated by dividing moles of VP1 per sample by 360 (72 pentamers per virion × 5 VP1 molecules per pentamer) and by the tissue volume (in μL/mm^3^). Linear regression, significance, and graphic display were generated in Prism 5 (GraphPad Software, Inc., La Jolla, CA).

## Results

### Case report and pathology

The patient’s clinical history and disease course have been reported [4, 21] [22]. Briefly, a 70-year-old man with 4 years of efalizumab treatment for psoriasis experienced progressive cognitive decline and was diagnosed with PML after detection of JCV in CSF. Efalizumab was stopped 3.5 weeks after symptom onset, and he was treated with plasma exchange (PLEX) 5 weeks after presentation with no clinical improvement. His neurologic status deteriorated, and he died 7.5 weeks after symptom onset. A brain-only autopsy confirmed the diagnosis of PML [21].

**Fig 2:**
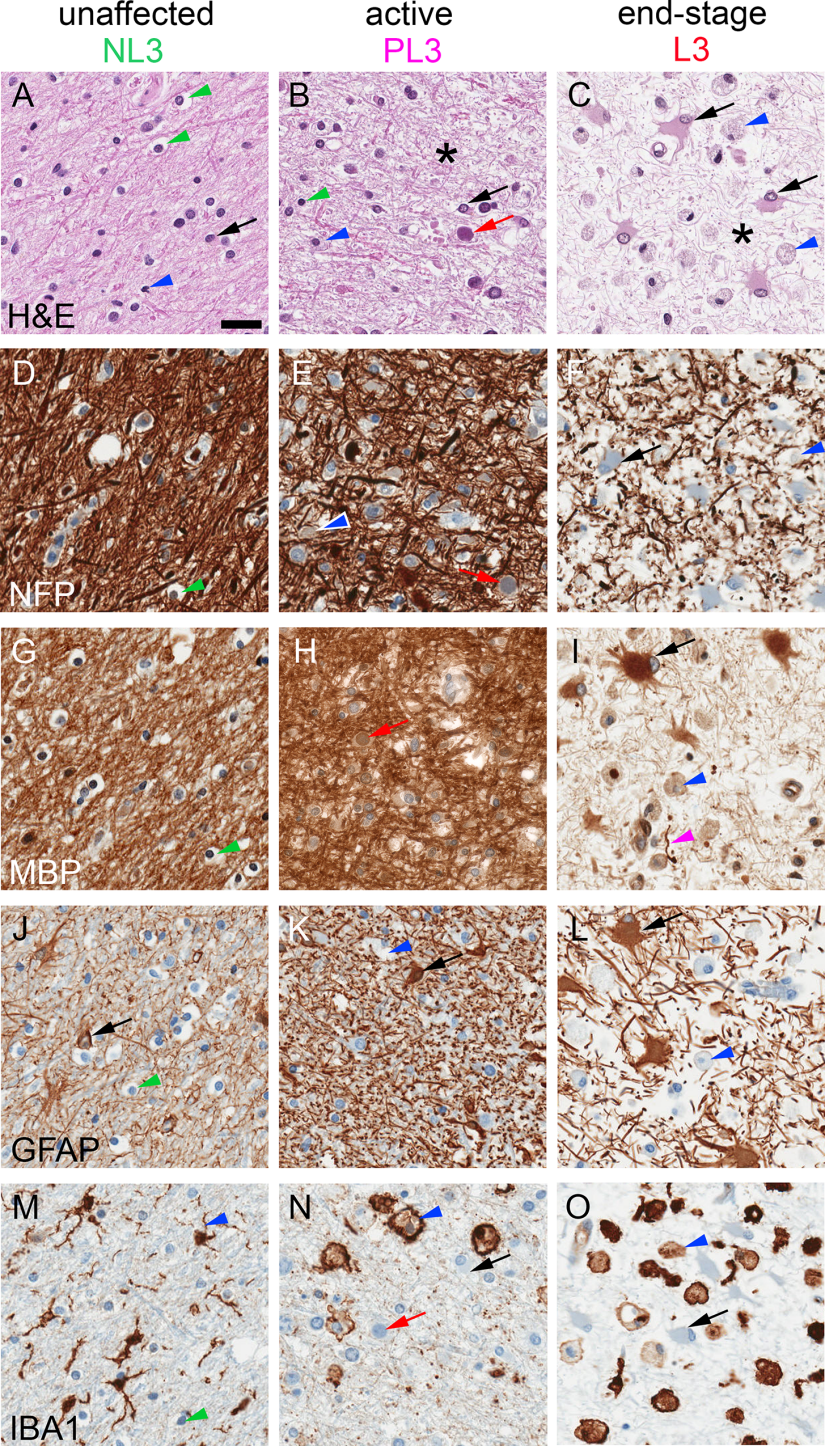
Spectrum of PML histopathology. H&E (A-C) and IHC (brown, with blue hematoxylin counterstain) for NFP to label neuronal processes (D-F), MBP to label myelin (G-I), GFAP to label astrocytes (black arrows) (J-L), and IBA1 to label microglia/macrophages (blue arrowheads) (M-O) from grossly unaffected, actively infected, and end-stage white matter (blocks NL3, PL3, and L3, respectively). NL3 (left column) had largely normal white matter composed of linear axons/myelin throughout the neuropil, oligodendrocytes with characteristic perinuclear “fried egg” halos (green arrowheads), astrocytes with thin and elongated processes, and microglia with short, thick processes. PL3 (middle column) showed active infection with viral nuclear inclusions (red arrows), hypertrophied astrocytes with thickened processes, swollen myelin, and some enlarged macrophages (this section also had foci of demyelination, not shown here). L3 (right column) had end-stage lesions with rare viral inclusions, reduced number and fragmentation of axons, residual axonal myelin (magenta arrowhead), and variable amounts of MBP within engorged macrophages and massively hypertrophied (“bizarre”) astrocytes. Asterisks in B and C designate thinning of neuropil secondary to axonal loss. Scale bar = 30 μm for all panels.

We excised grossly lesional (L), perilesional (PL), and adjacent nonlesional (NL) tissue samples from three brain areas, designated 1–3 (Methods and Fig 1). H&E staining and IHC stains to highlight neuronal axons/dendrites (neurofilament protein [NFP]), myelin (MBP), astrocytes (GFAP), and microglia/macrophages (IBA1) revealed the spectrum of PML pathology with regard to destruction of endogenous tissue, evidence of viral infection (enlarged oligodendrocytes with glassy, eosinophilic nuclear inclusions), altered cellular composition and morphology, and loss of axons and myelin (Fig 2; Table 1). NL3, corresponding to a brain region without apparent abnormality on T2-weighted magnetic resonance imaging (MRI) 1.5 week prior to death (Fig 1), showed largely normal white matter histology with rare viral inclusions; PL3 had active viral infection with patchy demyelination, many viral inclusions and enlarged, reactive astrocytes and microglia; and L3 had end-stage pathology with rare viral inclusions, severe axonal loss, reduced axonal myelin, and consisted predominantly of characteristic gemistocytic astrocytes and debris-laden macrophages without viral cytopathic changes (Fig 2; Table 1). In contrast to NL3, NL1 and NL2 showed active infection and areas of demyelination that were not evident grossly (Table 1), but originated from brain regions associated with white matter hyperintensities on MRI that can be seen in PML lesions [23] (Fig 1). In spite of the patient having peripheral CD4+ and CD8+ T lymphocyte counts within reference ranges 1 month prior to death (~1 week prior to PLEX) [4], lymphocytic infiltrates in the sections analyzed were scant: rare lymphocytes were restricted to perivascular Virchow-Robin spaces, with few to no lymphocytes in the lesional neuropil [21], consistent with residual efalizumab-associated reduction of lymphocyte trafficking and/or activation.

**Table 1:** Qualitative histopathologic features of each tissue block.

Block / location	Tissue composition	Lesion progression	Viral inclusions	Cellular composition	Myelin abundance	Features
L1 / L parieto-occipital	W	1, 2	1, 2	M, A, O	0, 1	“Wave” of infection
L2 / R inferior temporal	W	2	0	M, A	0	Severe demyelination
L3 / L anterior parietal	W + G	1, 2	1, 2	M, A, O, N	0, 1	Active infection abuts gray matter
PL1 / L parieto-occipital	W	0, 1	1	M, A, O	2	Early-stage infection
PL2 / R inferior temporal	W + G	1, 2	1, 2	M, A, N	1	End-stage infection abuts gray matter
PL3 / L anterior parietal	W	1, 2	2	M, A, O, N	0, 1	Patchy demyelination
NL1 / L parieto-occipital	W + G	0, 1, 2	2	M, A, O, N	1, 2	Targetoid, coalescent, and confluent active lesions
NL2 / R inferior temporal	W + G	1	2	M, A, O, N	1, 2	Targetoid, coalescent, and confluent active lesions
NL3 / L anterior parietal	W + G	0	0	M, A, O, N	2	Nearly normal-appearing white matter

Indicated tissue blocks were scored for PML histopathology as follows: Tissue composition: W, white matter, G, gray matter. Lesion progression: 0, unaffected/early lesions; 1, predominantly active lesions; 2, end-stage lesions. Viral inclusions: 0, absent to rare; 1, infrequent but evident; 2, abundant. Cellular composition: M, microglia/macrophages; A, astrocytes; O, oligodendroglia; N, neurons. Myelin abundance: 0, little to no myelin remaining; 1, partial myelin loss; 2, most myelinated axons intact.

### JCV quantitation and sequence analysis

**Fig 3:**
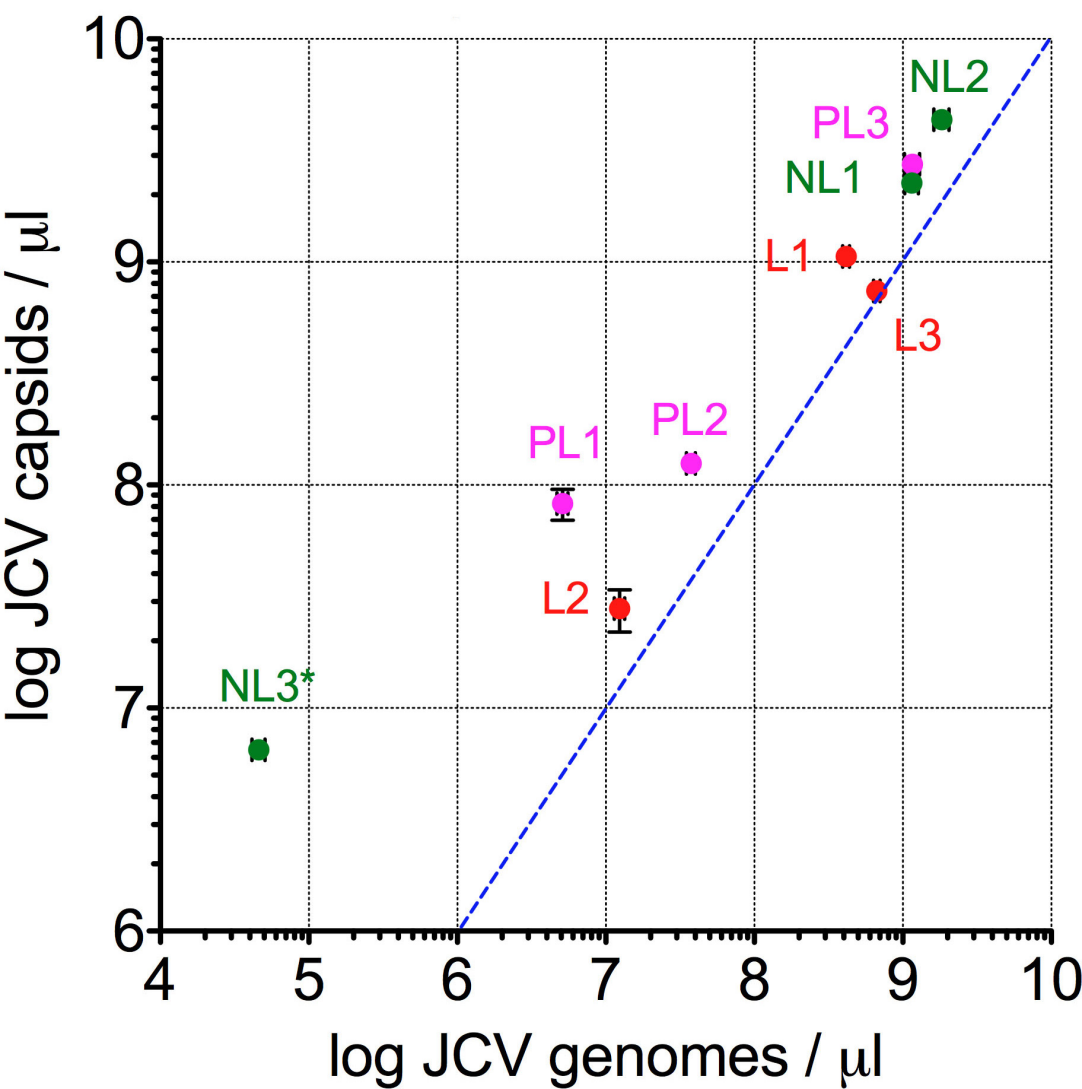
Quantification of JCV DNA and VP1 capsid protein. Log-log plot of the concentration of JCV genomes vs capsids per microliter tissue for all 9 PML blocks (Table 2). Data are means ± standard deviation based on triplicate measurements. Blue dashed line indicates theoretical relationship for one capsid per viral genome. Linear regression reveals a positive relationship with *r*^2^ = 0.96, *p*<0.0001 (regression line not shown). Asterisk designates the uncertainty of the protein measurement of VP1 in NL3, which was below the assay’s lower limit of quantitation (not shown).

To determine JCV concentrations in each tissue block, quantitative PCR for viral DNA and mass spectrometry to detect selected viral and host proteins were performed on formalin fixed paraffin embedded (FFPE) tissue slices of defined thickness, with tissue area quantified using whole slide image analysis (Table 2). Among the nine samples, there was a strong positive linear correlation (*r*^2^ = 0.96, *p*<0.0001) between viral DNA and capsid concentrations, the latter calculated from measurements of VP1 protein abundance (Fig 3; Table 2). Although reduced efficiency of DNA extraction from FFPE vs. frozen tissue might explain why sections from 7/9 blocks had ≤3-fold molar excess of viral capsids over viral genomes, our data are consistent with coordinate production of viral DNA and capsids during productive infection in vivo.

**Table 2:** Quantitation of virus components from each tissue block.

Block / location	Tissue area, mm^2^	Cells per μL tissue	Viral DNA per μL tissue	Viral capsids per μL tissue	Genome equivalents
L1 / L parieto-occipital	28.8	8.14E + 03	4.16E + 08	1.06E + 09	5.1E + 04
L2 / R inferior temporal	25.6	5.65E + 03	1.24E + 07	2.79E + 07	2.2E + 03
L3 / L anterior parietal	30.5	7.55E + 03	6.69E + 08	7.41E + 08	8.9E + 04
PL1 / L parieto-occipital	17.9	1.34E + 04	5.12E + 06	8.26E + 07	3.8E + 02
PL2 / R inferior temporal	22.4	6.44E + 03	3.75E + 07	1.25E + 08	5.8E + 03
PL3 / L anterior parietal	25.8	6.81E + 03	1.16E + 09	2.74E + 09	1.7E + 05
NL1 / L parieto-occipital	26.8	9.93E + 03	1.15E + 09	2.26E + 09	1.2E + 05
NL2 / R inferior temporal	23.0	2.14E + 04	1.83E + 09	4.35E + 09	8.5E + 04
NL3 / L anterior parietal	32.3	9.77E + 03	4.61E + 04	*6.49E + 06	4.72E + 00

Viral capsids were calculated from VP1 mass spectrometry measurements. Genome equivalents = viral genomes/cellular genomes in each block. Numbers, with the exception of areas, are expressed in scientific notation.

* indicates value below assay’s lower limit of quantitation.

**Fig 4:**
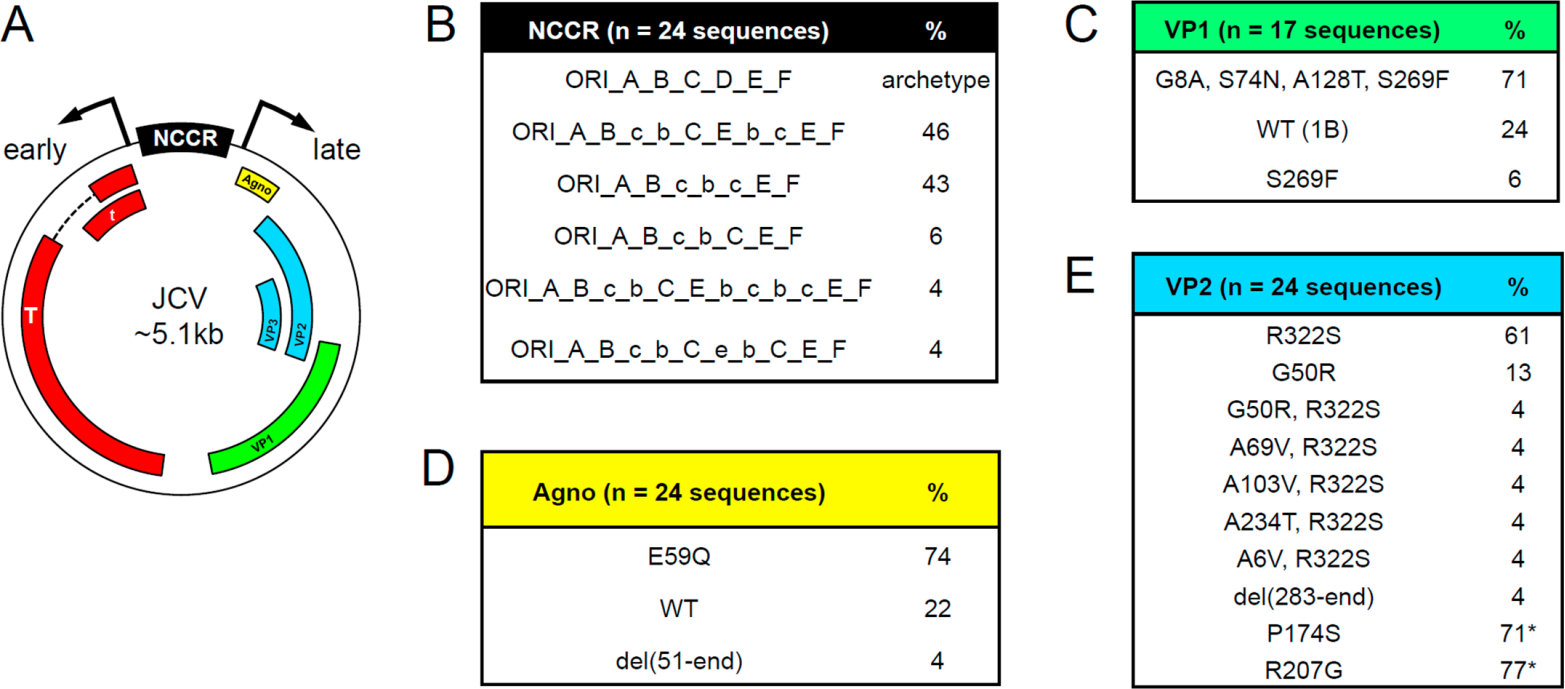
JCV sequence analysis. (A) Schematic of circular viral DNA showing sites of transcription initiation and directions of early and late transcription (arrows) adjacent to the NCCR. Regions encoding Early T-Ag (T and t) and late agnoprotein (Agno) and VP1, VP2, and VP3 proteins are indicated by colored boxes. (B) Summary of NCCR variants, with the archetype NCCR shown for reference consisting of an origin of replication (ORI) followed by intact sequence blocks A-F. The percent of sequences showing the schematized rearrangements, duplications, and/or mutant/deleted sequence blocks (indicated by lower case letters) are listed. (C) VP1 variants identified. WT (1B) = wild type, strain 1B. (D) Agnoprotein variants identified; del(51-end) = deletion of C-terminal 21 amino acids. (E) VP2 variants identified; del(283-end) = deletion of C-terminal 60 amino acids. P174S and R207G were identified in the VP1 amplicon, which overlaps the C-terminal region of VP2/3, with asterisks designating percent of VP1 region clones that harbored the VP2 mutation. Representative DNA sequences and alignments are shown in S1 File, and have been deposited at NCBI under accession nos. KX216358-KX216371.

We were unable to generate adequate amplicons for viral DNA sequence by PCR from FFPE tissue; rather, we amplified ~2kb regions of JCV encompassing the NCCR through VP1 using PCR from frozen lesional tissue (see Methods). All virus sequences were JCV strain 1B or variants thereof (data not shown) [24]. We identified 5 related NCCR rearrangements, all of which had the common D box deletion as well as partial or complete duplication of B and C boxes (Fig 4B). In addition, 77% (13/17) of clones harbored a previously described VP1 mutation S269F that is commonly identified in PML brain tissue, the majority of which also carried the triple point mutation G8A V74N A128T; wild-type VP1 was also isolated (Fig 4C). The majority of Agnoprotein clones carried the missense mutation E59Q, with a minority having deleted the C-terminal 21 amino acids (Fig 4D). Previously undescribed in the literature, we also identified ten VP2 variants (among a total of 41 clones), the majority with the missense mutation R322S and/or carrying one of seven missense mutations or a C-terminal 60 aa deletion (Fig 4E). Several noncoding and nonsense coding point mutations were also identified (S1 File). The diversity of JCV sequences indicates significant viral sequence evolution due to ongoing viral replication.

### JCV dispersion correlates with early demyelination

**Fig 5:**
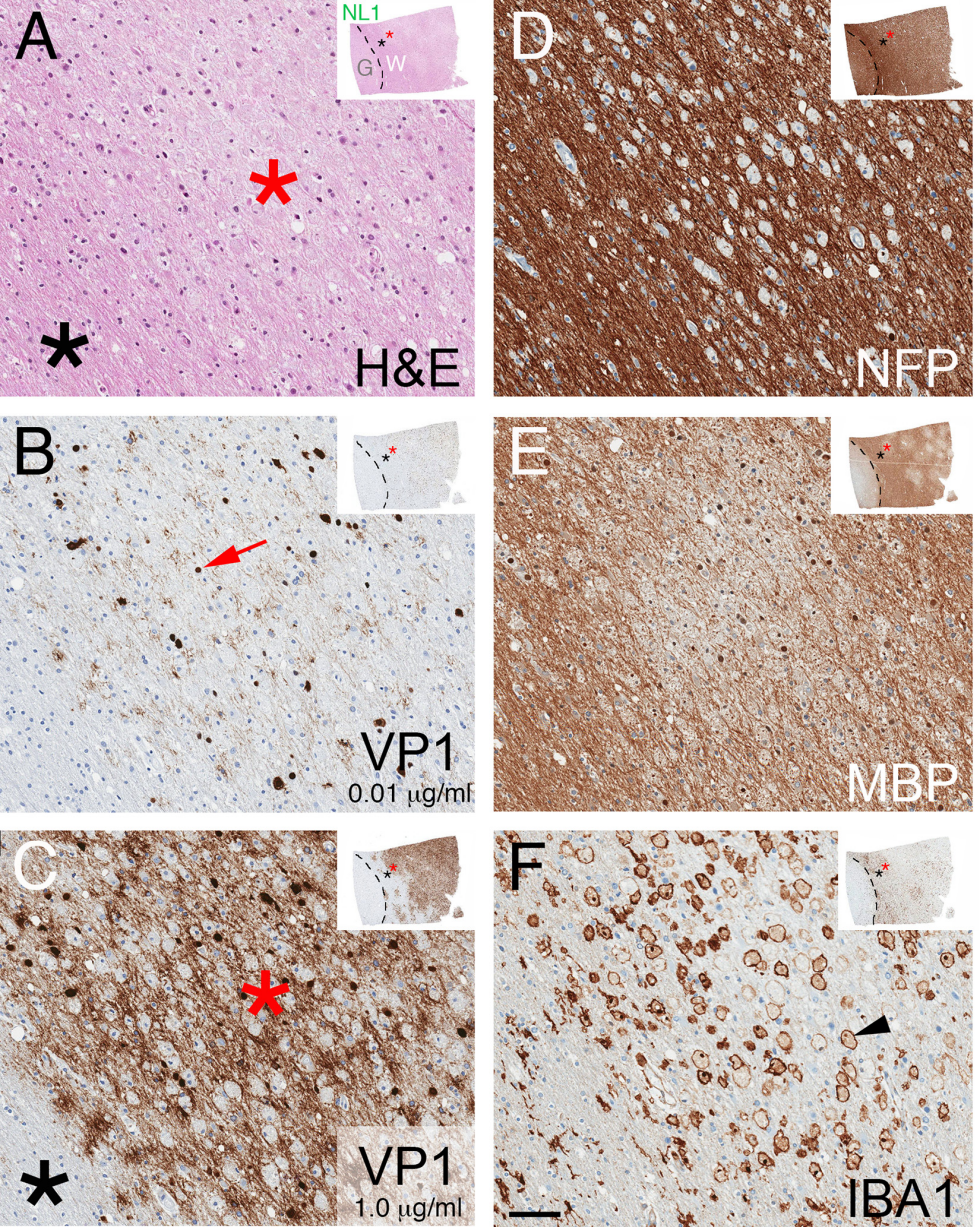
Histopathology of demyelinated foci. Adjacent sections of NL1 stained with H&E (A), IHC for VP1 using PAB597 at 0.01 μg/mL (B) or 1.0 μg/mL (C), NFP (D), MBP (E), or IBA1 (F). Thumbnail image in right upper corner of each panel shows low magnification image of stained section. Grey (G) / white (W) matter junction is denoted by a dashed line, and asterisks designate area around the focus of demyelination magnified in each panel. VP1 antibody at 0.01 μg/mL detects individually infected cells (red arrow, B), whereas 1 μg/mL additionally reveals broadly distributed VP1 (red asterisk, C), with no VP1 staining in nearby uninfected white matter (black asterisk) or gray matter. In demyelinated foci, there is no decrease in NFP stain (D) in areas with reduced MBP (E), and increased numbers of IBA+ engorged macrophages (arrowhead, F). Scale bar = 50 μm.

**Fig 6:**
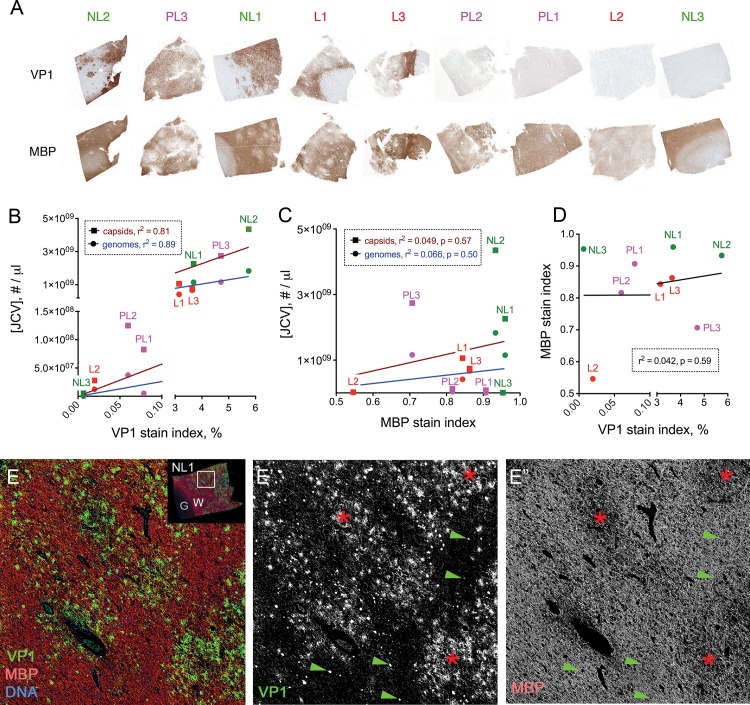
Spatial correlation of dispersed VP1 and early demyelination. A) Thumbnail IHC images of each block stained for VP1 (top row, left to right based on ranking from highest to lowest VP1 stain index) and MBP (bottom row). Linear plots of (B) JCV concentration vs. the percentage of section area stained darkly with VP1 at 0.01 μg/ml PAB597 (“VP1 stain index”), (C) JCV DNA concentration vs. the fraction of section area positive for MBP stain (“MBP stain index”) as a measure of residual myelin, and (D) MBP vs. VP1 stain indices. There were positive, statistically significant relationships between VP1 stain index and JCV genome or capsid concentrations (*p*<0.0001 for both lines in panel B), but not between MBP stain index and JCV concentrations (C), or between VP1 and MBP stain indices (D), with *r*^2^ and *p* values in boxes. E-E”) NL1 stained for VP1 (green; gray scale image of channel in panel E’), MBP (red; gray scale image of channel in panel E”), and DNA (blue) with fluorescence detection. White box on thumbnail image in right upper corner of panel E shows magnified area in E-E”, with gray (G) and white (W) matter regions designated. Demyelinated foci (red asterisks) are spatially coincident with dispersed VP1, whereas numerous VP1-positive cells (green arrowheads) can be seen in areas with intact myelin.

H&E stained sections of white matter with active infection showed pale neuropil associated with nuclear inclusions that stain by IHC using a limiting concentration of anti-VP1 antibody (e.g., section NL1 in Fig 5A and 5B) [25]. Two independent VP1 antibodies, when used at higher concentrations on such sections, in addition revealed dispersed viral immunoreactivity within areas of active infection, but no staining in adjacent tissue without PML pathology, nor in control (non-diseased), Alzheimer’s disease, or multiple sclerosis brain tissues (Fig 5C, S1 Fig, and not shown). The pale areas of white matter on H&E sections correspond to areas of demyelination, with reduced MBP but intact NFP and activated (IBA1+) macrophages in proximity to VP1+ cells (Fig 5A and 5D–5F). To investigate the spatial relationship between viral infection and demyelination, using whole slide image segmentation we quantified the fractional area (termed the “stain index”) of each block’s white matter with positive VP1 (using the lower concentration of VP1 antibody to label only infected cells) and MBP IHC staining as estimates of viral cytopathic effect and extent of demyelination, respectively (Fig 6A). Across the sample set there were positive, statistically significant correlations between VP1 stain index and either viral DNA or capsid concentrations (Fig 6B), but no correlations between MBP stain index and either of the measured virus concentrations (Fig 6C) or the VP1 stain index (Fig 6D). The lack of correlation between myelin abundance and viral cytopathic effect is likely due to the temporal heterogeneity of infection in the set of sampled tissues; e.g., a white matter section with abundant virus could have high or low amounts of residual myelin depending on the history of viral infection in that area of the brain. Confocal imaging of areas with newly demyelinated lesions (NL1, NL2) co-stained for MBP and VP1 showed a consistent association between foci of demyelination and dispersed VP1, but no relationship between demyelination and individual VP1+ infected cells (Fig 6E–6E”). Such correlation was less evident in more advanced and end-stage lesions, which also had dispersed virus but extensive loss of myelin and axons (e.g., blocks PL3, L1, and L3; not shown).

### JCV distributes within white matter myelin

**Fig 7:**
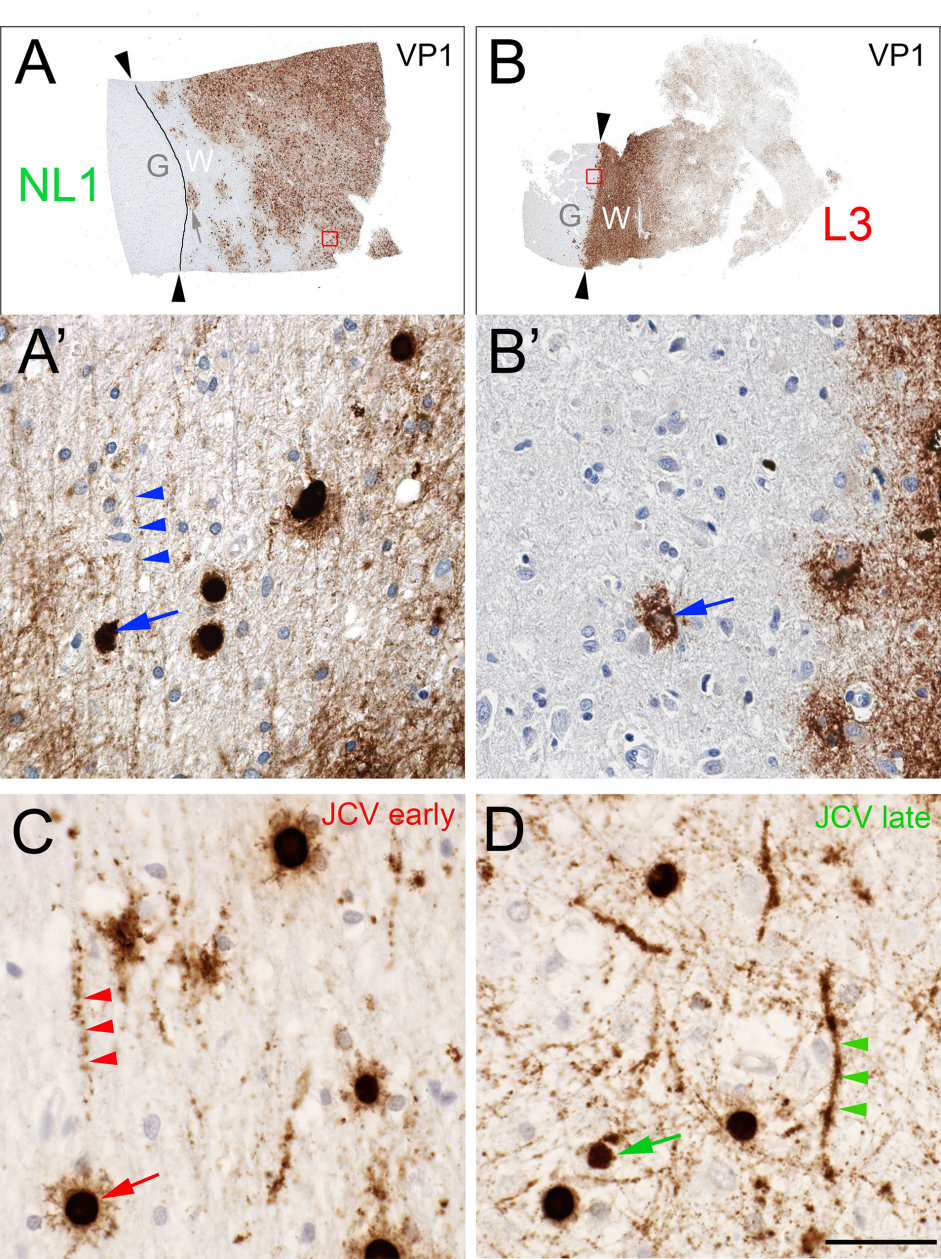
Distinct VP1 distributions in gray and white matter. VP1-stained NL1 (A) and L3 (B) with gray (G) and white (W) matter regions labeled, and black arrowheads (and line in A) demarcating the gray/white matter boundary. Red boxes in panels A and B show areas magnified below in panels A' and B', with blue arrows designating VP1-positive late stage (A’) or recently lysed (B’) cells. Note extensive linear VP1 throughout white matter (e.g., blue arrowheads in A') and little to no dispersed VP1 in gray matter. (C, D) JCV DNA distribution in PML white matter. RNAScope ISH for early (C) or Late (D) JCV probes labels individually infected cells with oligodendrocytic morphology (red and green arrows) as well as linear structures consistent with axons (red and green arrowheads). Scale bar = 5 mm in A, B; 100 μm in A', B'; C, D.

**Fig 8:**
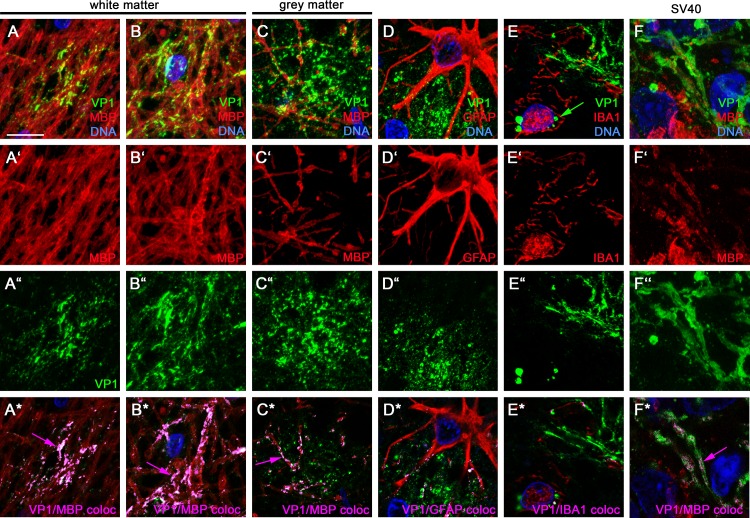
VP1 colocalizes with nearby myelin in recently lysed cells. Representative 5-μm confocal Z-axis stack images of regions of recent virus-induced cell lysis in otherwise intact white matter (A, B, D, E) or gray matter (C) of human PML, or white matter in an SIV-positive rhesus macaque with SV40 PML-like CNS disease (F), stained for VP1 (green in A-F, A″–F″, and A*-F*) and MBP (red in A-C, F, A′–C′, F′, and A*-C*, F*), GFAP (red in D, D′, D*), or IBA1 (red in E, E′, E*). A*–F* show representative colocalization (coloc) images from a single image from each Z-stack, with magenta pixels designating VP1/MBP colocalization (magenta arrows); Image quantitation of these representative Z-stack images revealed 89% and 86% of VP1 colocalized with MBP in white matter (A* and B*, respectively); 34% colocalized in gray matter (C*); and 13% and 2.9% of VP1 colocalized with GFAP or IBA1 (D* and E*, respectively). In SV40 PML-like disease (F), a subset of VP1 was dispersed in a linear pattern and colocalized with partially demyelinated MBP-positive axons (magenta arrow in F*). Occasional cytoplasmic VP1 viral aggregates were seen in cytoplasm of microglia (green arrow in E) or astrocytes, but nuclei of these cell types did not show VP1 positivity that would be indicative of productive infection. Scale bar in panel A = 10 μm.

**Fig 9:**
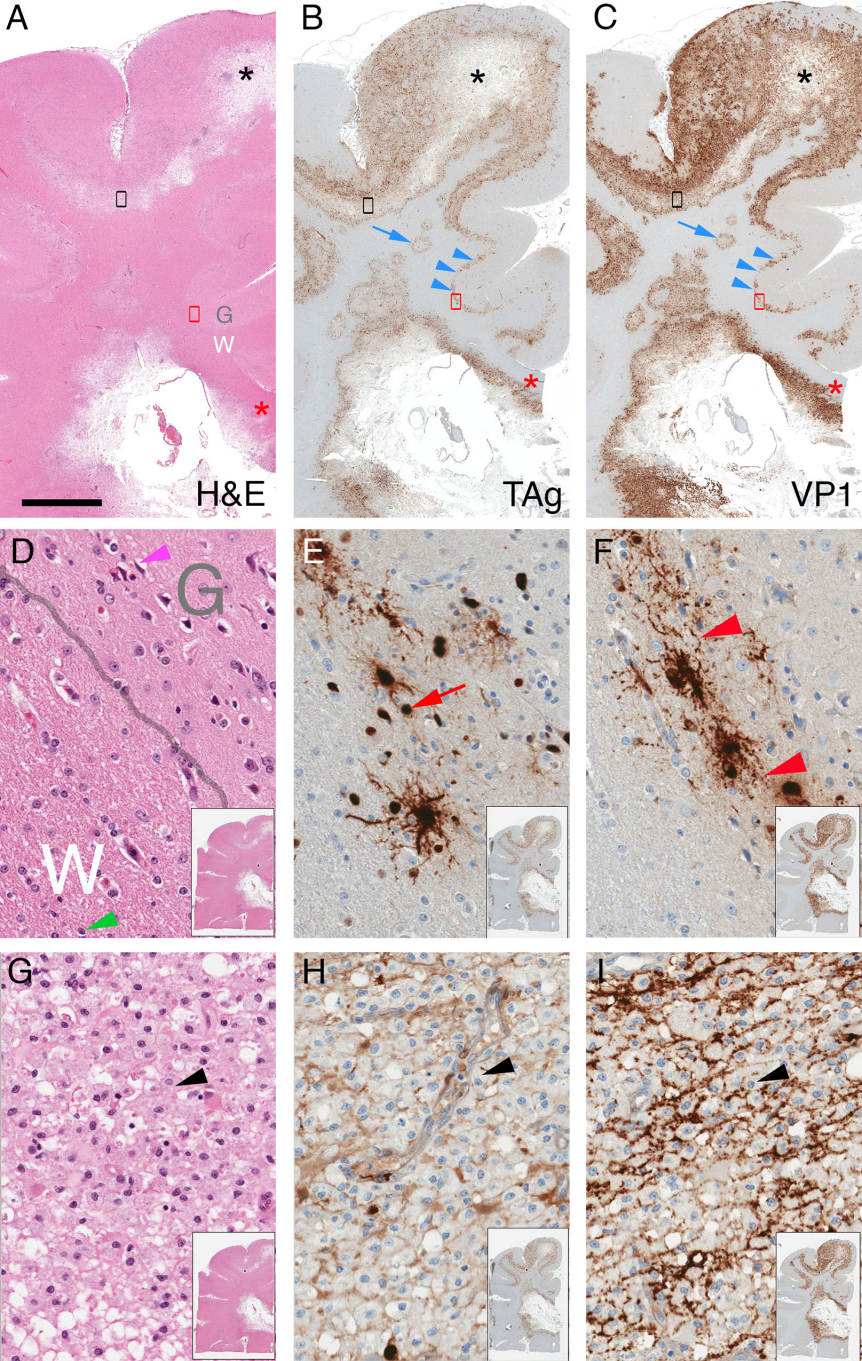
Histopathology and virus protein distributions in SV40 PML-like disease. Representative section of macaque cerebrum was fixed, stained for H&E (A, D, G), TAg (B, E, H), or VP1 (C, F, I). (A-C) Black asterisks mark cortical area with severe demyelination, and red asterisks mark corpus callosum. Gray (G) and white (W) matter regions are designated, with areas in red and black boxes in A-C magnified in D–F and G–I, respectively. Note foci of infection within white matter (blue arrow) and tracking along gray-white junction (blue arrowheads). (D-F) Magnified area of infected gray-white matter junction, showing actively infected area with TAg-positive cells (red arrow) and VP1-positive infected cells with dispersed VP1 (red arrowhead). (G–I) End-stage lesion showing dispersed TAg and VP1 amid sheets of macrophages (black arrowhead). Inset images in lower right corners of D-I show low magnification view of entire tissue section. Scale bar: 5 mm in A–C, 100 μm in D–I.

PML predominantly affects white matter, but cerebellar and cortical gray matter can be involved [17, 26–28]. VP1 distributions in blocks NL1 and L3 illustrated JCV’s preference for white matter over gray matter. Using the higher concentration of either VP1 antibody, block NL1 showed confluent white matter PML lesions with VP1 dispersed throughout much of the white matter; one lesion abutted but did not transgress the white/gray matter junction (e.g., Fig 7A). Block L3 had severe demyelination, with dispersed VP1 throughout the subcortical white matter, but the most intense VP1 staining sharply abutted the entire white/gray matter junction, with no VP1 in gray matter except within and adjacent to rare infected satellite cells (Fig 7B). In all blocks with VP1+ cells, dispersed VP1 was linearly distributed in white matter, but to a lesser extent in gray matter (Fig 7A’ and 7B’), suggesting a preferential association of dispersed JCV with white matter myelinated axons. In addition to labeling individually infected cells, a linear distribution of dispersed virus was also observed using RNAscope in situ hybridization to detect viral nucleic acid (Fig 7C and 7D; see Methods) [20]. Confocal microscopy of fluorescent-stained sections confirmed extensive colocalization of MBP with dispersed VP1 in white matter, readily seen adjacent to recently lysed cells in otherwise intact white matter (Fig 8A and 8B). Although some VP1/MBP colocalization was seen adjacent to recently lysed grey matter lesions, there was far less VP1/MBP colocalization in gray matter than in white matter (Fig 8C). Neither VP1 antibodies nor JCV ISH labeled cells with astrocytic or microglial morphology, and while rare cells with astroglial or microglial morphology were seen in association with VP1 aggregates, dispersed VP1 showed minimal to no association or colocalization with GFAP-positive astrocytic processes (Fig 8D) or IBA1-positive microglial / macrophage processes (Fig 8E). VP1 of SV40, a related polyomavirus that infects monkeys, was also widely dispersed in an SIV-infected rhesus macaque who died from PML-like leukoencephalopathy (Fig 9). Dispersed VP1 was observed throughout lesional tissue, particularly along the gray/white matter junction, at the expanding edge of circular lesions, and in end-stage lesions (Fig 9). Confocal microscopy revealed partial SV40 VP1/MBP colocalization along myelinated axons (Fig 8F), but to a lesser extent than JCV VP1 in the human case. These findings suggest that polyomavirus spreads via the myelin sheath throughout white matter, but that viral spread is dramatically impeded at the white/gray matter junction as virus encounters gray matter myelin.

### JCV infection is spatially dynamic, traveling in “waves” through white matter

**Fig 10:**
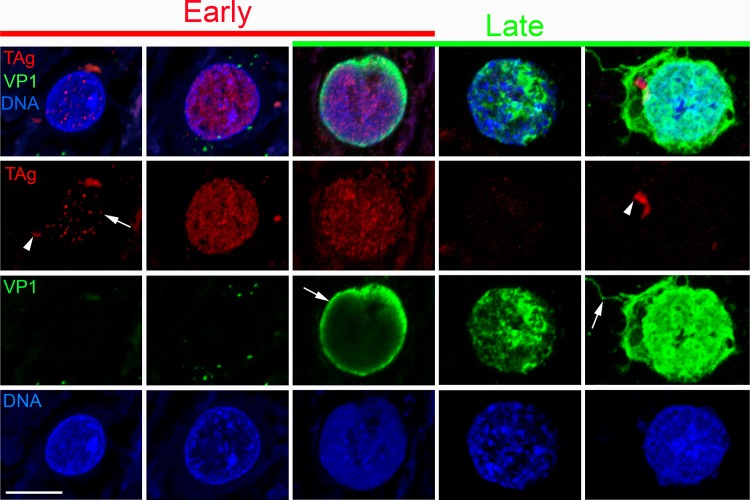
JCV protein distributions during the viral life cycle. Representative confocal images of JCV-infected white matter oligodendrocytes at various stages of infection, stained for TAg (red), VP1 (green), and DNA (blue), from early to late stages (left to right). Early infection (left two columns) is marked by nuclear foci of TAg (arrow), which increase in number as the nucleus enlarges. Cells transitioning between early and late stages (middle column) show abundant nuclear TAg foci with peripheral nuclear VP1 denoting early viral assembly (arrow). At late stages of infection (right two columns), TAg is absent, and VP1 fills the cytoplasm and proximal extent of some cell processes but does not appear to extend to processes that ensheathe nearby axons (arrow). Cytoplasmic lipofuscin (arrowheads) in some cells was captured with TAg staining with AF594 detection (red channel). Scale bar = 10 μm.

**Fig 11:**
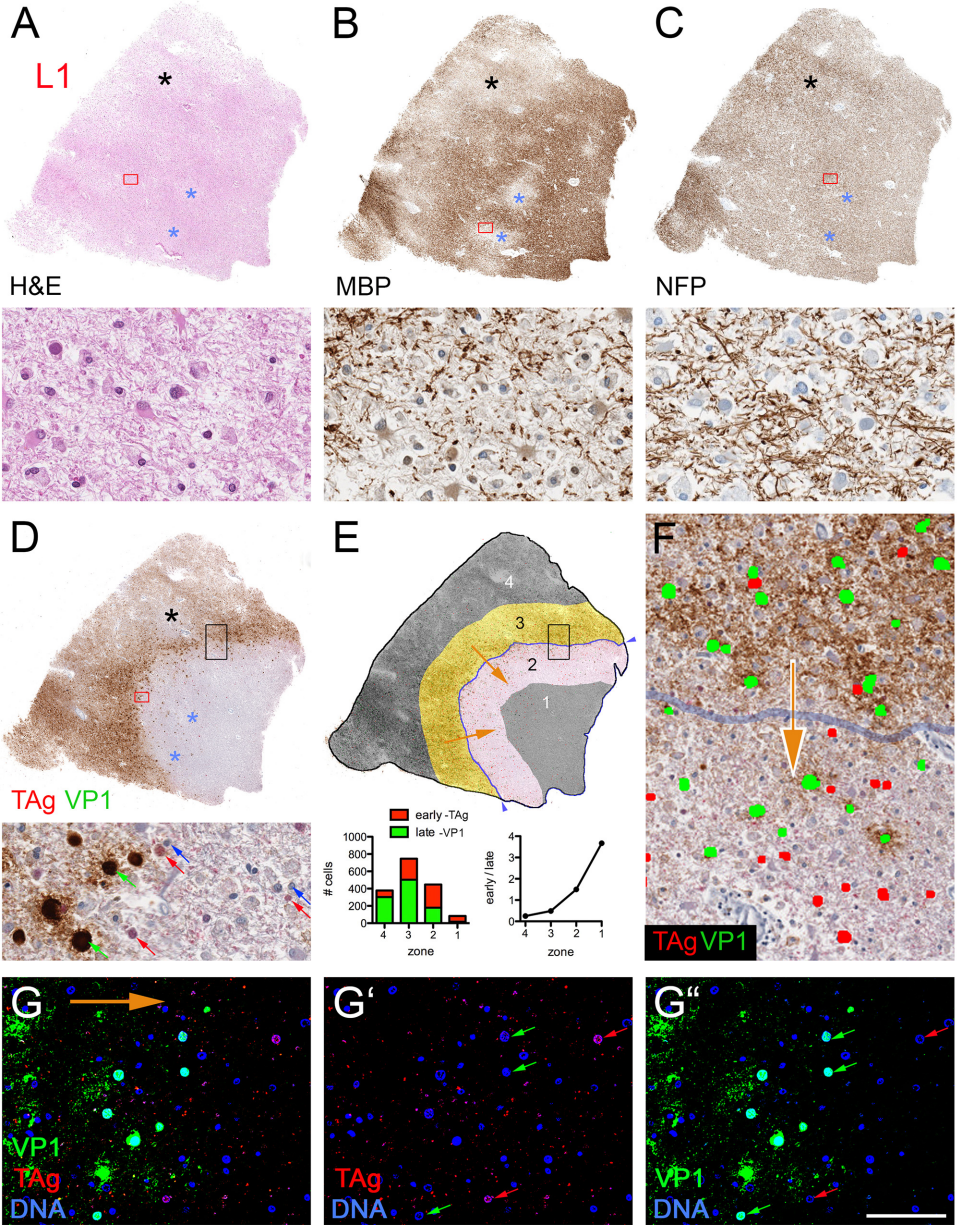
White matter “wave” of JCV infection. (A-D) Top of each panel shows low magnification views of sequential sections of block L1, stained for H&E (A) or indicated IHCs (B-D), with higher magnification of region designated by red box shown in bottom of each panel. Black asterisk designates confluent area of demyelination behind wave; blue asterisks designate two foci of MBP pallor ahead of VP1 wave, indicative of recent demyelination. Panel D is a dual IHC, revealing TAg with alkaline phosphatase/fast red detection (red arrows) and VP1 with HRP/DAB detection (green arrow). (E) Schematic of four zones (1–4) relative to wave front of dispersed virus (blue line demarcated by blue arrowheads at the edge of the section) overlying the tissue image in panel D following manual segmentation of TAg-positive cells (red dots) and VP1-positive cells (green dots). Orange arrows indicate putative direction of wave movement through the section. Graphs show cell counts for TAg (red) and VP1 (green) (bottom left), and ratio of early (TAg positive) to late (VP1 positive) infected cells as a function of zone (bottom right). (F) Magnified area of wave front indicated by black box in panels D and E, with TAg-positive cells false-colored red, VP1-positive cells false-colored green, and wave front (gray line) inferred to be progressing in the direction indicated by orange arrow. G–G"–confocal image at wave front stained for TAg (red), VP1 (green), and DNA (blue), with orange arrow showing direction of wave. (Punctate, non-nuclear red stain represents nonspecific autofluorescence due to endogenous lipofuscin.) Scale bar in G" = 2 mm in top panels of A-E, 50 μm in bottom panels of A–D and in G–G" and 70 μm in F.

The dynamics of infection at the time of death in the efalizumab PML brain were inferred by staining for early (TAg) and late (VP1) markers of viral infection. Confocal imaging of dual stained sections revealed subcellular distributions of TAg and VP1 that correlated with alterations in cell morphology at early and late stages of viral infection (Fig 10). Block L1, consisting entirely of white matter, revealed staining patterns consistent with infection advancing in a wave-like manner through this region at the time of death. H&E of this section revealed patchy pallor; the pale areas were confirmed as areas of marked demyelination based on reduced MBP but uniform NFP stain on serial sections (asterisks, Fig 11A–11C). Single and double IHC for TAg and VP1 revealed partially overlapping zones of stained nuclei, with a prominent ~0.5–1-mm wide arc of dispersed VP1 spanning the section (Fig 11D). We used image analysis and manual cell counts to quantify the number of TAg- and VP1-positive cells within four tissue zones: zone 1, in front of the wave; zones 2 and 3, adjacent 1-mm–wide zones that flank the “wave front” of dispersed VP1; and zone 4, in the wake of the wave (Fig 11E and 11F). The directional movement of infection predicts more cells in the early stage of infection ahead of the wave of dispersed VP1, and more late-stage cells behind the wave. This was indeed observed: the ratio of TAg/VP1-positive cells progressively decreased in each zone from the front to the rear of the wave, from 3.67 in zone 1 to 0.25 in zone 4 (Fig 11E). Double IF / confocal microscopy for TAg and VP1 unambiguously identified cells in early, mid, and later stages of infection, respectively, confirming many TAg-positive cells ahead of the VP1-positive cells ahead of the wave front (Fig 11G).

## Discussion

We report genomic, proteomic, and immunopathologic characterization of JC virus infection in a case of efalizumab-associated PML. Our data demonstrate that virus concentrations vary over several orders of magnitude in different regions of brain with progressive PML, with maximal JCV tissue concentrations approximating those previously reported in PML brain tissue (>10^5^ virus genomes per cellular genome [21, 29]). Notably, two of the three highest JCV concentrations were in grossly “non-lesional” tissue blocks NL1 and NL2, each piece of tissue appearing grossly normal to the unaided eye, but both associated with T2 MRI hyperintensities ~1.5 week prior to death, suggesting that MRI might be a sensitive marker of active infection. Minimal PML histopathology and the lowest JCV concentrations were found in NL3, from a region of the brain not associated with MRI abnormality. As expected, the three grossly lesional sections (L1-L3) demonstrated predominantly end-stage histopathology, but each had JCV concentrations one to two orders of magnitude lower than the blocks with the highest concentrations; these lesional blocks also had lower amounts of dispersed VP1 staining and JCV DNA by in situ hybridization than NL1 and NL2 (Fig 3, and data not shown). The observed peak tissue JCV concentrations in this case might be near the maximum achievable by a polyomavirus in a CNS infection because host inflammatory response in the brain was minimal to absent, despite drug withdrawal and plasma exchange therapy in an attempt to reverse the immunosuppressive effects of efalizumab [21].

JCV sequences isolated from lesional PML brain tissue revealed sequence diversity consistent with dynamic viral evolution during infection. While genomic alterations in the NCCR, VP1, and agnoprotein have been previously detected in PML, this is the first identification to our knowledge of diverse mutations in the minor capsid protein VP2. Interestingly, we only detected rearranged NCCRs, but both wild-type and mutant VP1. This finding is consistent with NCCR rearrangements being necessary for early brain infection [12, 30], whereas VP1 mutations arise as infection commences in the brain, as has been observed in human PML cases as well as in an animal model for JCV infection in immunocompromised, myelin-deficient mice whose white matter was reconstituted with human glial progenitors [19]. The effects of the numerous VP2 mutations we identified on JCV viral life cycle and PML pathogenesis are unknown.

The extent of JCV dispersion and VP1/myelin association seen in this case of PML was unexpected, and has not been previously reported in the literature. We do not know whether our findings are common in PML or whether they are specific to this case or a subset of cases. The paucity of lymphocytic infiltrates coupled with abundant virus indicates that the patient’s immune responses in the CNS were depressed, in spite of normal T lymphocyte counts and PLEX to accelerate efalizumab clearance. Using independent methods to detect JCV DNA and protein, we documented a correlation between dispersed virus and demyelination at advancing fronts of white matter infection, as well as extensive colocalization between virus and white matter myelin sheath. Electron microscopy previously revealed dispersed virions in PML tissue, both in extracellular aggregates–many myelin-associated–as well as within myelin sheath lamellae [31, 32], and one prior report used in-situ PCR to reveal JCV nucleic acid amidst myelin [33]. That we also observed dispersed VP1 and VP1/MBP colocalization in a case of SV40-associated PML-like CNS disease in a rhesus macaque suggests that viral association with the myelin sheath and demyelination might be a general feature of CNS polyomavirus infections.

The mechanism by which JCV spreads throughout white matter in PML remains unknown. MRI-defined lesions can expand radially, travel along white matter tracts, or rapidly involve entire regions of white matter [34]. If JCV only spread locally following lysis of a productively infected cell, in a cell-by-cell fashion, one might expect to see “rings” (corresponding to spheres in three dimensions) of TAg-positive nuclei surrounding foci of recently lysed cells, but this was not seen in our efalizumab case. Rather, recently lysed cells were often immediately adjacent to uninfected, TAg-negative oligodendrocytes, suggesting that in addition to virus diffusion between cells, a second mechanism is responsible for spread of infection in the CNS. Our data raise the hypothesis that a specific association of dispersed JCV with the myelin sheath biases the spread of infection along white matter axon tracts and promotes demyelination.

The oligodendrocyte has long been considered to be the predominant, productively infected cell type in most cases of human PML [35]. However, recent data from a mouse/human chimera model of JCV infection suggest that astrocytes might be the primary infected cell type in early PML [19]. Differences between end-stage human autopsy brain and an animal model brain tissue are numerous, and our data in this case do not support astrocytes as significantly contributing to productive infection or transmitting virus throughout the white matter. While astroctyes with productive JCV infection have been documented in human PML [16], in our nine FFPE tissue blocks we only observed a single TAg-positive VP1-negative cell with astrocytic morphology that expressed GFAP; this cell was in an area with end stage infection, severe axonal loss, and little to no residual axonal myelin (data not shown). In our efalizumab case, any JCV/MBP colocalization could simply be a consequence of JCV remaining associated with residual oligodendrocyte membranes on axons following lysis of infected oligodendrocytes. Indeed, the presence of virions within myelin lamellae as viewed by electron microscopy has been used to argue that JCV infection spreads predominantly via an intracellular route [36, 37]. Although VP1 antibodies labeled proximal oligodendrocyte processes in very late stage infected cells (i.e., markedly enlarged TAg-negative cells with strong VP1 positivity in nucleus and cytoplasm, Fig 10), there was little to no colocalization between myelin and these VP1-labeled processes at this late stage of cellular infection (data not shown); the “scattershot” pattern of VP1 distribution following lysis of infected cells (e.g., Fig 8A–8E) consists of discontinuous, heterogeneous VP1 aggregates that do not resemble the remnants of cellular processes but rather appear to be dispersed extracellularly, and were consistently associated with nearly all axons in the vicinity of the lysed cell. The marked JCV VP1/MBP colocalization we observed around recently lysed cells could also be a nonspecific consequence of neuronal or axonal injury to nearby neuropil, but a predilection of virus for axonal myelin was confirmed by minimal colocalization of VP1 with astrocytic or microglial cells or their processes. Indeed, in some areas, axons with extensive myelin loss had linear aggregates of VP1 partially to completely replacing discontinuous myelin (Fig 9B and 9F), suggesting that as viral aggregates accumulate on the myelin sheath, they replace myelin.

**Fig 12:**
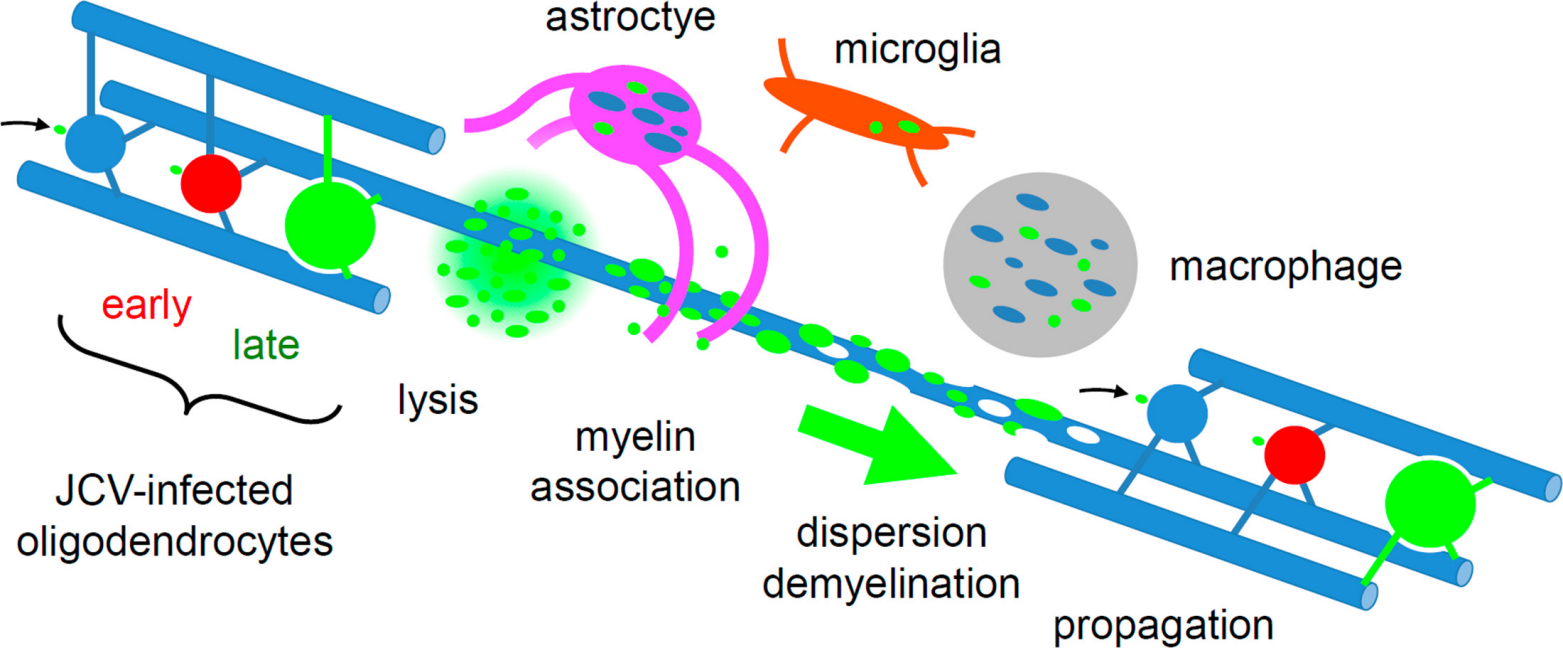
Model for PML propagation in white matter from this case. JCV (green dot) infects myelinating oligodendrocytes (blue, with processes ensheathing axons), which progress from early (red, TAg) to late (green, VP1) infection. Some virus spreads intracellularly within oligodendroglial processes toward the myelin sheath. Infected oligodendrocytes lyse and most viral aggregates associate with nearby myelin, leading to extracellular virus dispersion along white matter tracts (large green arrow), demyelination, and propagation by infection of distant oligodendrocytes (small black arrow). Variable amounts of myelin (blue) and virus are seen in association with astrocytes (magenta), microglia (orange), and macrophages (gray).

As schematized in Fig 12, our data from this case suggest at least two mechanisms by which JCV can spread in PML: 1) local, intracellular spread of virus via oligodendrocyte processes, and 2) longer range, extracellular dispersion of virus along white matter tracts via myelinated axons. A local, intracellular mechanism might account for radial (centrifugal) expansion of early lesions, whereas the latter mechanism might be more prominent when PML spreads over longer distances along the grid-like array of white matter tracts that comprise the mammalian brain [38].

Two tissue blocks captured putative “waves” of infection moving through tissue: one whose movement appeared to be halted at the white/gray matter junction (L3, Fig 7B), and another coursing through partially demyelinated white matter at the time of death (L1, Fig 11). The waves we observed might be similar to the gradient of demyelination across PML lesions observed by Webster and colleagues [36]. However, we did not observe, as they did, an obvious gradient of macrophages (not shown), perhaps because of the patient’s underlying disease (psoriasis) or state of immunosuppression at the time of death. The marked preference of JCV for white matter axons over grey matter axons in this case suggests an intrinsic heterogeneity in the distribution of responsible molecules targeted by JCV, possibly due to intra-axonal patterning mechanisms [39], chemical heterogeneity of myelin along axons [40], or other mechanisms.

In summary, our analysis of JC virus sequence analysis, abundance, and distribution in PML brain tissue from an efalizumab-treated psoriasis patient implicates the white matter myelin sheath as both an “enabler” and a “victim” in the intracerebral spread of PML. Whether polyomavirus dispersion via white matter myelinated axons is common feature of demyelination and axonal destruction in human PML merits further investigation.

## Supporting Information

S1 Fig VP1 Antibody Specifity. Representative images of brain sections from a control patient with multiple sclerosis (MS brain, panels A, C, E, and G) or from this PML patient (block NL2, with abundant dispersed virus, panels B, D, F, and H), stained with VP1 mouse monoclonal antibody (mAb) PAB597 (panels A-D) or VP1 rabbit polyclonal antibody (pAb) ab53977 (panels E-H; see Methods), detected with DAB (brown), and counterstained with hematoxylin (light blue). At 0.01 μg/ml, PAB597 does not label any cells (e.g., satellite cells / oligodendrocyte, green arrowhead), vasculature (yellow arrowhead), or neuropil (red asterisk) in the MS brain (panel A), but does label the nucleus of individually infected cells (red arrow) and, weakly, neuropil (red asterisk) of the PML brain section (panel B) while leaving many cells including small uninfected oligodendrocytes unlabeled (green arrowhead). At 1 μg/ml, PAB597 shows very weak nonspecific diffuse neuropil staining (asterisk) but no specific cell labeling of the MS brain (panel C), whereas the PML brain section (panel D) shows extensive neuropil staining (red asterisk) that spares the nuclei of uninfected cells (green arrowhead) and vasculature (yellow arrowhead), consistent with the staining being specific for VP1. Similarly, at 1:8,000 dilution, ab53977 does not label any structures in the MS brain (panel E) but on PML brain (panel F) shows a similar staining pattern as the mouse mAb PAB597; at 1:2,000 dilution, ab53977 labels MS brain (panel G) and PML brain (panel H) in a very similar pattern as PAB597 at 1 μg/ml. Non-diseased and Alzheimer’s brain sections showed a similar staining patterns as the MS brain, and all control brains showed appropriate staining of GFAP and IBA1 for astrocytes and microglia, respectively, indicating the control tissues were competent for detection of antigens with the IHC method (not shown).
(DOCX)

S1 File JCV DNA Sequences. (DOCX)
