# Supplementary material for: JC Polyomavirus Abundance and Distribution in Progressive Multifocal Leukoencephalopathy (PML) Brain Tissue Implicates Myelin Sheath in Intracerebral Dissemination of Infection
Source: PLoS One. 2016 May 18;11(5):e0155897. doi: 10.1371/journal.pone.0155897 (PMC4871437; doi:10.1371/journal.pone.0155897)
Supplement: S1 Fig — Representative images of brain sections from a control patient with multiple sclerosis (MS brain, panels A, C, E, and G) or from this PML patient (block NL2, with abundant dispersed virus, panels B, D, F, and H), stained with VP1 mouse monoclonal antibody (mAb) PAB597 (panels A-D) or VP1 rabbit polyclonal antibody (pAb) ab53977 (panels E-H; see Methods), detected with DAB (brown), and counterstained with hematoxylin (light blue). At 0.01 μg/ml, PAB597 does not label any cells (e.g., satellite cells / oligodendrocyte, green arrowhead), vasculature (yellow arrowhead), or neuropil (red asterisk) in the MS brain (panel A), but does label the nucleus of individually infected cells (red arrow) and, weakly, neuropil (red asterisk) of the PML brain section (panel B) while leaving many cells including small uninfected oligodendrocytes unlabeled (green arrowhead). At 1 μg/ml, PAB597 shows very weak nonspecific diffuse neuropil staining (asterisk) but no specific cell labeling of the MS brain (panel C), whereas the PML brain section (panel D) shows extensive neuropil staining (red asterisk) that spares the nuclei of uninfected cells (green arrowhead) and vasculature (yellow arrowhead), consistent with the staining being specific for VP1. Similarly, at 1:8,000 dilution, ab53977 does not label any structures in the MS brain (panel E) but on PML brain (panel F) shows a similar staining pattern as the mouse mAb PAB597; at 1:2,000 dilution, ab53977 labels MS brain (panel G) and PML brain (panel H) in a very similar pattern as PAB597 at 1 μg/ml. Non-diseased and Alzheimer’s brain sections showed a similar staining patterns as the MS brain, and all control brains showed appropriate staining of GFAP and IBA1 for astrocytes and microglia, respectively, indicating the control tissues were competent for detection of antigens with the IHC method (not shown). (DOCX) [file pone.0155897.s001.docx]

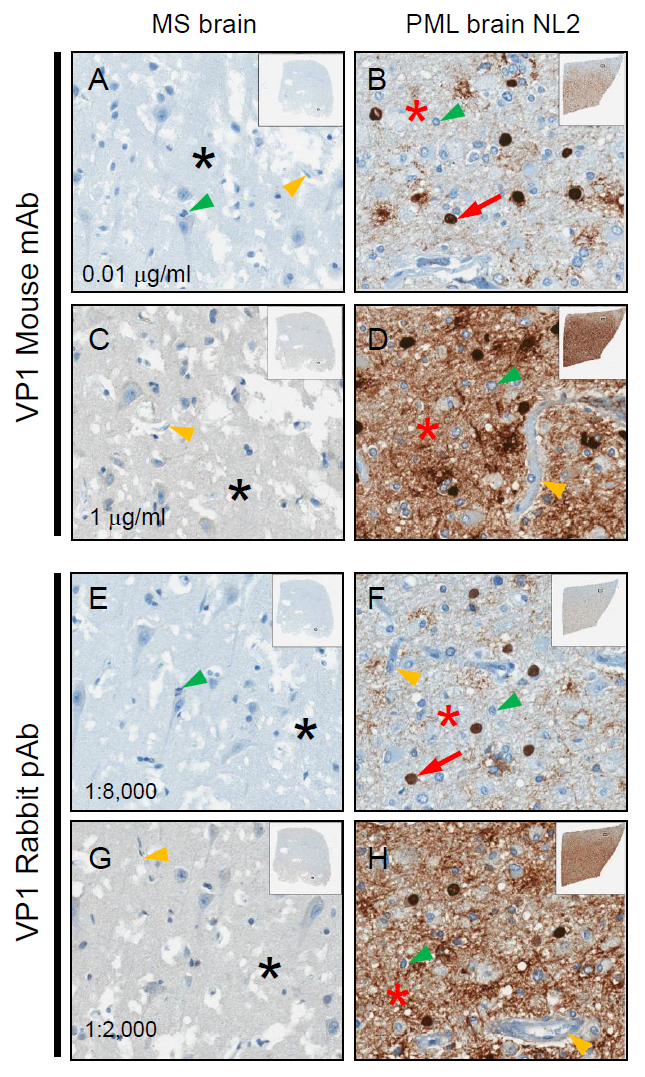


**Supplemental Figure.** VP1 antibody control stains. Representative images of brain sections from a control patient with multiple sclerosis (MS brain, panels A, C, E, and G) or from this PML patient (block NL2, with abundant dispersed virus, panels B, D, F, and H), stained with VP1 mouse monoclonal antibody (mAb) PAB597 (panels A-D) or VP1 rabbit polyclonal antibody (pAb) ab53977 (panels E-H; see Methods), detected with DAB (brown), and counterstained with hematoxylin (light blue). At 0.01 µg/ml, PAB597 does not label any cells (e.g., satellite cells / oligodendrocyte, green arrowhead), vasculature (yellow arrowhead), or neuropil (red asterisk) in the MS brain (panel A), but does label the nucleus of individually infected cells (red arrow) and, weakly, neuropil (red asterisk) of the PML brain section (panel B) while leaving many cells including small uninfected oligodendrocytes unlabeled (green arrowhead)**.** At 1 µg/ml, PAB597 shows very weak nonspecific diffuse neuropil staining (asterisk) but no specific cell labeling of the MS brain (panel C), whereas the PML brain section (panel D) shows extensive neuropil staining (red asterisk) that spares the nuclei of uninfected cells (green arrowhead) and vasculature (yellow arrowhead), consistent with the staining being specific for VP1. Similarly, at 1:8,000 dilution, ab53977 does not label any structures in the MS brain (panel E) but on PML brain (panel F) shows a similar staining pattern as the mouse mAb PAB597; at 1:2,000 dilution, ab53977 labels MS brain (panel G) and PML brain (panel H) in a very similar pattern as PAB597 at 1 µg/ml. Non-diseased and Alzheimer’s brain sections showed a similar staining patterns as the MS brain, and all control brains showed appropriate staining of GFAP and IBA1 for astrocytes and microglia, respectively, indicating the control tissues were competent for detection of antigens with the IHC method (not shown).
