# Supplementary material for: JC Polyomavirus Abundance and Distribution in Progressive Multifocal Leukoencephalopathy (PML) Brain Tissue Implicates Myelin Sheath in Intracerebral Dissemination of Infection
Source: PLoS One. 2016 May 18;11(5):e0155897. doi: 10.1371/journal.pone.0155897 (PMC4871437; doi:10.1371/journal.pone.0155897)
Supplement: S1 File — (DOCX) [file pone.0155897.s002.docx]

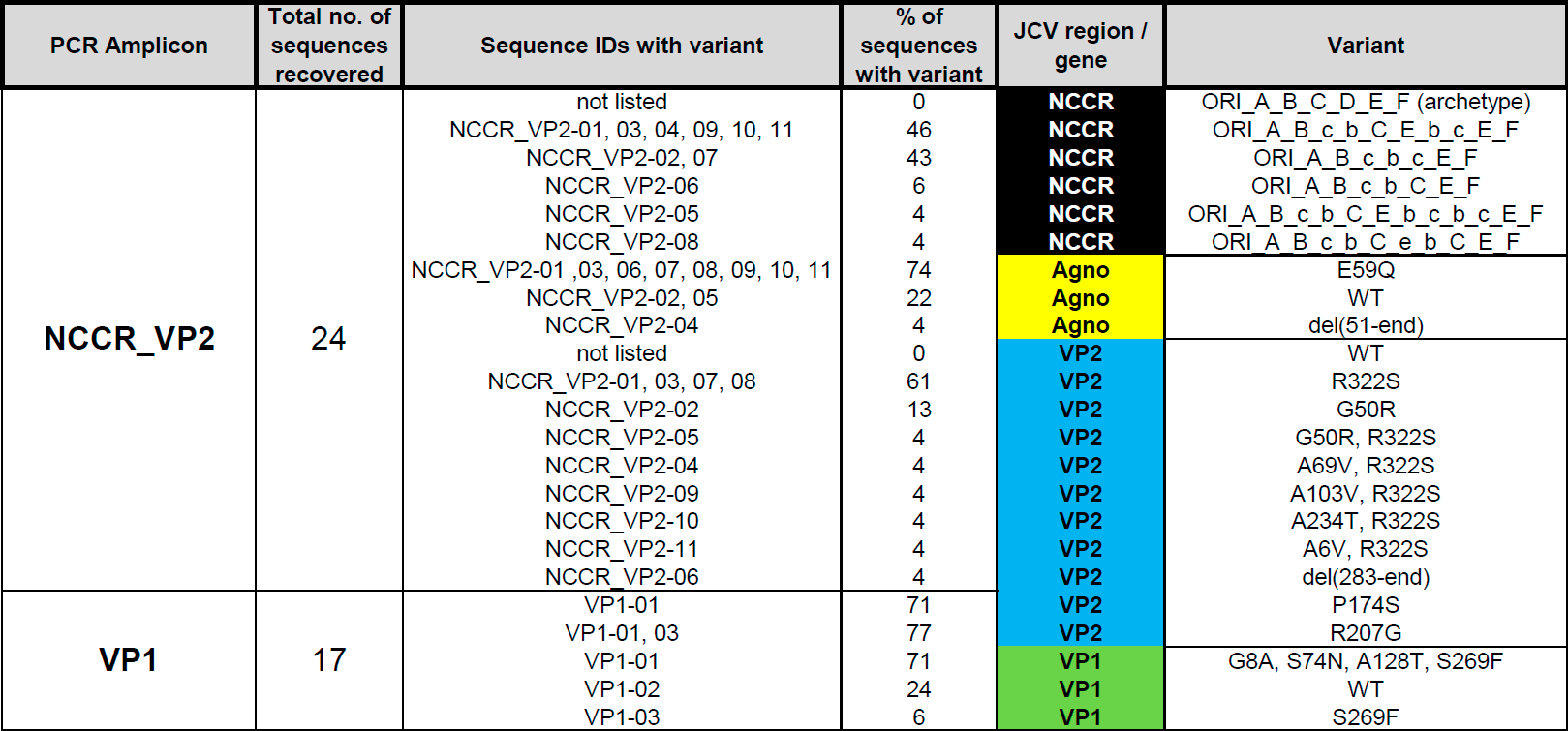


Table shows summary of DNA sequencing data from the efalizumab/PML brain that predicted variants either in the NCCR or changes in Agno, VP2, or VP1 amino acid sequences, with reference to alignments of representative “NCCR_VP2” or “VP1” PCR amplicon sequences that follow on pages 2-11 and 12-15 of this document, respectively. The variants, corresponding sequence IDs, number of total clones and percent of cloned sequences with each variant, and region / genes with each variant are indicated.

In the “NCCR_VP2” amplicon alignment, the NCCR is at nucleotides 58-441, the 71 aa agnoprotein is encoded by nucleotide nos. 442-654; and the 344 aa VP2 protein is encoded by nucleotide nos. 691-1722 (the VP3 coding region overlaps with the C-terminal 225 amino acids of VP2, with the VP3 initiator methionine at nucleotide no. 1048). The NCCR_VP2 amplicon also includes N-terminal amino acids of the VP1 protein, with the VP1 start codon at nucleotide no. 1633, 5’ of the VP2/VP3 stop codon at nucleotide no. 1723.

In the “VP1” amplicon alignment, the initiator methionine for VP3 is at nucleotide no. 23, the 354 aa VP1 protein is encoded by nucleotide nos. 609-1673, and the VP2/3 coding region is terminated by a stop codon at nucleotide 698.

Notes about alignments:

1. Italicized terminal nucleotides in each alignment are linker cloning sequences, not part of the JCV genome.
2. Nucleotide gaps are marked with a dash.
3. In the NCCR region of “NCCR_VP2” alignment, variable or missing nucleotides are designated in red, and “NCCR block” row indicates sequences of intact terminal NCCR sequence blocks A_B and E_F.
4. Nucleotide variants that create silent coding mutations or are outside coding regions are designated in red, with an asterisk marking the position with the change; nucleotides variants that create specific missense or frameshift/deletion mutations are indicated, with reading frame in the mutant sequence underlined.
5. Initiator methionine ATG codons are colored for each encoded protein, and stop codons used are colored dark red.
6. The Agno mutation del(51-end) is predicted to remove amino acid R51, fusing K50 with out of frame sequence encoding the six amino acids DRDTVV-stop; this clone also carries the Agno E59Q mutation, but it is out of frame.
7. The VP2 mutation del(283-end) is predicted to remove amino acid P283, fusing A282 to a stop codon at nucleotide no. 1716. This intragenic deletion is also predicted to eliminate the N-terminal 27 amino acids of VP1, including the VP1 initiator methionine. The next intact 3’ VP1 methionine residue is M48. This clone thus lacks both C-terminal sequence of VP2/3 and N terminal sequence of VP1.


Each of the representative sequences have been deposited at NCBI under the accession nos. xxxxx-yyyyy.

Efalizumab/PML brain DNA “NCCR-VP2” sequences

Nucleotide 1 50

NCCR_VP2-01 (1) *GAATTCGCCCTT*CCTCCACGCCCTTACTACTTCTGAGTAAGCTTGGAGGC

NCCR_VP2-02 (1) *GAATTCGCCCTT*CCTCCACGCCCTTACTACTTCTGAGTAAGCTTGGAGGC

NCCR_VP2-05 (1) *GAATTCGCCCTT*CCTCCACGCCCTTACTACTTCTGAGTAAGCTTGGAGGC

NCCR_VP2-06 (1) *GAATTCGCCCTT*CCTCCACGCCCTTACTACTTCTGAGTAAGCTTGGAGGC

NCCR_VP2-07 (1) *GAATTCGCCCTT*CCTCCACGCCCTTACTACTTCTGAGTAAGCTTGGAGGC

NCCR_VP2-08 (1) *GAATTCGCCCTT*CCTCCACGCCCTTACTACTTCTGAGTAAGCTTGGAGGC

NCCR_VP2-04 (1) *GAATTCGCCCTT*CCTCCACGCCCTTACTACTTCTGAGTAAGCTTGGAGGC

NCCR_VP2-03 (1) *GAATTCGCCCTT*CCTCCACGCCCTTACTACTTCTGAGTAAGCTTGGAGGC

NCCR_VP2-09 (1) *GAATTCGCCCTT*CCTCCACGCCCTTACTACTTCTGAGTAAGCTTGGAGGC

NCCR_VP2-10 (1) *GAATTCGCCCTT*CCTCCACGCCCTTACTACTTCTGAGTAAGCTTGGAGGC

NCCR_VP2-11 (1) *GAATTCGCCCTT*CCTCCACGCCCTTACTACTTCTGAGTAAGCTTGGAGGC

51 100

NCCR_VP2-01 (51) GGAGGCGGCCTCGGCCTCCTGTATATATAAAAAAAAGGGAAGGTAGGGAG

NCCR_VP2-02 (51) GGAGGCGGCCTCGGCCTCCTGTATATATAAAAAAAAGGGAAGGTAGGGAG

NCCR_VP2-05 (51) GGAGGCGGCCTCGGCCTCCTGTATATATAAAAAAAAGGGAAGGTAGGGAG

NCCR_VP2-06 (51) GGAGGCGGCCTCGGCCTCCTGTATATATAAAAAAAAGGGAAGGTAGGGAG

NCCR_VP2-07 (51) GGAGGCGGCCTCGGCCTCCTGTATATATAAAAAAAAGGGAAGGTAGGGAG

NCCR_VP2-08 (51) GGAGGCGGCCTCGGCCTCCTGTATATATAAAAAAAAGGGAAGGTAGGGAG

NCCR_VP2-04 (51) GGAGGCGGCCTCGGCCTCCTGTATATATAAAAAAAAGGGAAGGTAGGGAG

NCCR_VP2-03 (51) GGAGGCGGCCTCGGCCTCCTGTATATATAAAAAAAAGGGAAGGTAGGGAG

NCCR_VP2-09 (51) GGAGGCGGCCTCGGCCTCCTGTATATATAAAAAAAAGGGAAGGTAGGGAG

NCCR_VP2-10 (51) GGAGGCGGCCTCGGCCTCCTGTATATATAAAAAAAAGGGAAGGTAGGGAG

NCCR_VP2-11 (51) GGAGGCGGCCTCGGCCTCCTGTATATATAAAAAAAAGGGAAGGTAGGGAG

NCCR block AAAAAAAAAAAAAAAAAAAAAAAAAAAAAAAAAAAABBBBBBB

101 150

NCCR_VP2-01 (101) GAGCTGGCTAAAACTGGATGGCTGCCAGCCAAGCATGAGCTCATGGCTAA

NCCR_VP2-02 (101) GAGCTGGCTAAAACTGGATGGCTGCCAGCCAAGCATGAGCTCAT------

NCCR_VP2-05 (101) GAGCTGGCTAAAACTGGATGGCTGCCAGCCAAGCATGAGCTCATGGCTAA

NCCR_VP2-06 (101) GAGCTGGCTAAAACTGGATGGCTGCCAGCCAAGCATGAGCTCATGGCTAA

NCCR_VP2-07 (101) GAGCTGGCTAAAACTGGATGGCTGCCAGCCAAGCATGAGCTCATGGCTAA

NCCR_VP2-08 (101) GAGCTGGCTAAAACTGGATGGCTGCCAGCCAAGCATGAGCTCATGGCTAA

NCCR_VP2-04 (101) GAGCTGGCTAAAACTGGATGGCTGCCAGCCAAGCATGAGCTCATGGCTAA

NCCR_VP2-03 (101) GAGCTGGCTAAAACTGGATGGCTGCCAGCCAAGCATGAGCTCATGGCTAA

NCCR_VP2-09 (101) GAGCTGGCTAAAACTGGATGGCTGCCAGCCAAGCATGAGCTCATGGCTAA

NCCR_VP2-10 (101) GAGCTGGCTAAAACTGGATGGCTGCCAGCCAAGCATGAGCTCATGGCTAA

NCCR_VP2-11 (101) GAGCTGGCTAAAACTGGATGGCTGCCAGCCAAGCATGAGCTCATGGCTAA

NCCR block BBBBBBBBBBBBBBBBCCCCCCCCCCCCCCCCCCCCCCCCCCCC------

151 200

NCCR_VP2-01 (151) AACTGGATGGCTGCCAGCCAAGCATGAGCTCATACCTAGGGAGCCAACCA

NCCR_VP2-02 (145) --------------------------------------------------

NCCR_VP2-05 (151) AACTGGATGGCTGCCAGCCAAGCATGAGCTCATACCTAGGGAGCCAACCA

NCCR_VP2-06 (151) AACTGGATGGCTGCCAGCCAAGCATGAGCTCATACCTAGGGAGCCAACCA

NCCR_VP2-07 (151) AACTGGATGGCTGCCAGCCAAGCATGAGCTCATG--------GCCAACCA

NCCR_VP2-08 (151) AACTGGATGGCTGCCAGCCAAGCATGAGCTCATACCTAGGGAGCCAACCA

NCCR_VP2-04 (151) AACTGGATGGCTGCCAGCCAAGCATGAGCTCATACCTAGGGAGCCAACCA

NCCR_VP2-03 (151) AACTGGATGGCTGCCAGCCAAGCATGAGCTCATACCTAGGGAGCCAACCA

NCCR_VP2-09 (151) AACTGGATGGCTGCCAGCCAAGCATGAGCTCATACCTAGGGAGCCAACCA

NCCR_VP2-10 (151) AACTGGATGGCTGCCAGCCAAGCATGAGCTCATACCTAGGGAGCCAACCA

NCCR_VP2-11 (151) AACTGGATGGCTGCCAGCCAAGCATGAGCTCATACCTAGGGAGCCAACCA

NCCR block ---------------------------------*----------------

201 250

NCCR_VP2-01 (201) GCTGACAGCCAAACA-----------------------------------

NCCR_VP2-02 (145) --------------------------------------------------

NCCR_VP2-05 (201) GCTGACAGCCAAACAAAGCACAAGGCTGGCTAAAACTGGATGGCTGCCAG

NCCR_VP2-06 (201) GCTGACAGCCAAACA-----------------------------------

NCCR_VP2-07 (193) GCTGACAGCCAAACA-----------------------------------NCCR_VP2-08 (201) GCTGACAGCCAAACA-----------------------------------

NCCR_VP2-04 (201) GCTGACAGCCAAACA-----------------------------------NCCR_VP2-03 (201) GCTGACAGCCAAACA-----------------------------------NCCR_VP2-09 (201) GCTGACAGCCAAACA-----------------------------------NCCR_VP2-10 (201) GCTGACAGCCAAACA-----------------------------------

NCCR_VP2-11 (201) GCTGACAGCCAAACA-----------------------------------

NCCR block ---------------***********************************

251 300

NCCR_VP2-01 (216) ----------------AAGCACAAGGC-------------TGGCTAAAAC

NCCR_VP2-02 (145) ----------------AATCACAAGTAAA----CAGGAGCTGGCTAAAAC

NCCR_VP2-05 (251) CCAAGCATGAGCTCATAATCACAAGTAAA----CAGGAGCTGGCTAAAAC

NCCR_VP2-06 (216) ----------------AAGCACAA--------------------------

NCCR_VP2-07 (208) ----------------AAGCACAA--------------------------

NCCR_VP2-08 (216) ----------------AAGCACAATGGGAAGTGGAGCTCATGGCTAAAAC

NCCR_VP2-04 (216) ----------------AAGCACAAGGC-------------TGGCTAAAAC

NCCR_VP2-03 (216) ----------------AAGCACAAGGC-------------TGGCTAAAAC

NCCR_VP2-09 (216) ----------------AAGCACAAGGC-------------TGGCTAAAAC

NCCR_VP2-10 (216) ----------------AAGCACAAGGC-------------TGGCTAAAAC

NCCR_VP2-11 (216) ----------------AAGCACAAGGC-------------TGGCTAAAAC

NCCR block ****************--*-----****-*****--****----------

301 350

NCCR_VP2-01 (237) TGGATGGCTGCCAGCCAAGCATGAGCTCATG--------GCCAACCAGCT

NCCR_VP2-02 (175) TGGATGGCTGCCAGCCAAGCATGAGCTCATAA-----------------T

NCCR_VP2-05 (297) TGGATGGCTGCCAGCCAAGCATGAGCTCATAA-----------------T

NCCR_VP2-06 (224) --------------------------------------------------

NCCR_VP2-07 (216) --------------------------------------------------

NCCR_VP2-08 (250) TGGATGGCTGCCAGCCAAGCATGAGCTCATACCTAGGGAGCCAACCAGCT

NCCR_VP2-04 (237) TGGATGGCTGCCAGCCAAGCATGAGCTCATG--------GCCAACCAGCT

NCCR_VP2-03 (237) TGGATGGCTGCCAGCCAAGCATGAGCTCATG--------GCCAACCAGCT

NCCR_VP2-09 (237) TGGATGGCTGCCAGCCAAGCATGAGCTCATG--------GCCAACCAGCT

NCCR_VP2-10 (237) TGGATGGCTGCCAGCCAAGCATGAGCTCATG--------GCCAACCAGCT

NCCR_VP2-11 (237) TGGATGGCTGCCAGCCAAGCATGAGCTCATG--------GCCAACCAGCT

NCCR block ------------------------------**------------------

351 400

NCCR_VP2-01 (279) GACAGCCAAACAAAGCACAAGGGGAAGTGGAAAGCAGCCAAGGGAACATG

NCCR_VP2-02 (208) CACAAGTAAACAAAGCACAAGGGGAAGTGGAAAGCAGCCAAGGGAACATG

NCCR_VP2-05 (330) CACAAGTAAACAAAGCACAAGGGGAAGTGGAAAGCAGCCAAGGGAACATG

NCCR_VP2-06 (224) --------------------GGGGAAGTGGAAAGCAGCCAAGGGAACATG

NCCR_VP2-07 (216) --------------------GGGGAAGTGGAAAGCAGCCAAGGGAACATG

NCCR_VP2-08 (300) GACAGCCAAACAAAGCACAAGGGGAAGTGGAAAGCAGCCAAGGGAACATG

NCCR_VP2-04 (279) GACAGCCAAACAAAGCACAAGGGGAAGTGGAAAGCAGCCAAGGGAACATG

NCCR_VP2-03 (279) GACAGCCAAACAAAGCACAAGGGGAAGTGGAAAGCAGCCAAGGGAACATG

NCCR_VP2-09 (279) GACAGCCAAACAAAGCACAAGGGGAAGTGGAAAGCAGCCAAGGGAACATG

NCCR_VP2-10 (279) GACAGCCAAACAAAGCACAAGGGGAAGTGGAAAGCAGCCAAGGGAACATG

NCCR_VP2-11 (279) GACAGCCAAACAAAGCACAAGGGGAAGTGGAAAGCAGCCAAGGGAACATG

NCCR block *---E**EEEEEEEEEEEEEEEFFFFFFFFFFFFFFFFFFFFFFFFFFFF

401 450

NCCR_VP2-01 (329) TTTTGCGAGCCAGAGCTGTTTTGGCTTGTCACCAGCTGGCCATGGTTCTT

NCCR_VP2-02 (258) TTTTGCGAGCCAGAGCTGTTTTGGCTTGTCACCAGCTGGCCATGGTTCTT

NCCR_VP2-05 (380) TTTTGCGAGCCAGAGCTGTTTTGGCTTGTCACCAGCTGGCCATGGTTCTT

NCCR_VP2-06 (254) TTTTGCGAGCCAGAGCTGTTTTGGCTTGTCACCAGCTGGCCATGGTTCTT

NCCR_VP2-07 (246) TTTTGCGAGCCAGAGCTGTTTTGGCTTGTCACCAGCTGGCCATGGTTCTT

NCCR_VP2-08 (350) TTTTGCGAGCCAGAGCTGTTTTGGCTTGTCACCAGCTGGCCATGGTTCTT

NCCR_VP2-04 (329) TTTTGCGAGCCAGAGCTGTTTTGGCTTGTCACCAGCTGGCCATGGTTCTT

NCCR_VP2-03 (329) TTTTGCGAGCCAGAGCTGTTTTGGCTTGTCACCAGCTGGCCATGGTTCTT

NCCR_VP2-09 (329) TTTTGCGAGCCAGAGCTGTTTTGGCTTGTCACCAGCTGGCCATGGTTCTT

NCCR_VP2-10 (329) TTTTGCGAGCCAGAGCTGTTTTGGCTTGTCACCAGCTGGCCATGGTTCTT

NCCR_VP2-11 (329) TTTTGCGAGCCAGAGCTGTTTTGGCTTGTCACCAGCTGGCCATGGTTCTT

NCCR block FFFFFFFFFFFFFFFFFFFFFFFFFFFFFFFFFFFFFFFF-Agno

451 500

NCCR_VP2-01 (379) CGCCAGCTGTCACGTAAGGCTTCTGTGAAAGTTAGTAAAACCTGGAGTGG

NCCR_VP2-02 (308) CGCCAGCTGTCACGTAAGGCTTCTGTGAAAGTTAGTAAAACCTGGAGTGG

NCCR_VP2-05 (430) CGCCAGCTGTCACGTAAGGCTTCTGTGAAAGTTAGTAAAACCTGGAGTGG

NCCR_VP2-06 (304) CGCCAGCTGTCACGTAAGGCTTCTGTGAAAGTTAGTAAAACCTGGAGTGG

NCCR_VP2-07 (296) CGCCAGCTGTCACGTAAGGCTTCTGTGAAAGTTAGTAAAACCTGGAGTGG

NCCR_VP2-08 (400) CGCCAGCTGTCACGTAAGGCTTCTGTGAAAGTTAGTAAAACCTGGAGTGG

NCCR_VP2-04 (379) CGCCAGCTGTCACGTAAGGCTTCTGTGAAAGTTAGTAAAACCTGGAGTGG

NCCR_VP2-03 (379) CGCCAGCTGTCACGTAAGGCTTCTGTGAAAGTTAGTAAAACCTGGAGTGG

NCCR_VP2-09 (379) CGCCAGCTGTCACGTAAGGCTTCTGTGAAAGTTAGTAAAACCTGGAGTGG

NCCR_VP2-10 (379) CGCCAGCTGTCACGTAAGGCTTCTGTGAAAGTTAGTAAAACCTGGAGTGG

NCCR_VP2-11 (379) CGCCAGCTGTCACGTAAGGCTTCTGTGAAAGTTAGTAAAACCTGGAGTGG

501 550

NCCR_VP2-01 (429) AACTAAAAAAAGAGCTCAAAGGATTTTAATTTTTTTGTTAGAATTTTTGC

NCCR_VP2-02 (358) AACTAAAAAAAGAGCTCAAAGGATTTTAATTTTTTTGTTAGAATTTTTGC

NCCR_VP2-05 (480) AACTAAAAAAAGAGCTCAAAGGATTTTAATTTTTTTGTTAGAATTTTTGC

NCCR_VP2-06 (354) AACTAAAAAAAGAGCTCAAAGGATTTTAATTTTTTTGTTAGAATTTTTGC

NCCR_VP2-07 (346) AACTAAAAAAAGAGCTCAAAGGATTTTAATTTTTTTGTTAGAATTTTTGC

NCCR_VP2-08 (450) AACTAAAAAAAGAGCTCAAAGGATTTTAATTTTTTTGTTAGAATTTTTGC

NCCR_VP2-04 (429) AACTAAAAAAAGAGCTCAAAGGATTTTAATTTTTTTGTTAGAATTTTTGC

NCCR_VP2-03 (429) AACTAAAAAAAGAGCTCAAAGGATTTTAATTTTTTTGTTAGAATTTTTGC

NCCR_VP2-09 (429) AACTAAAAAAAGAGCTCAAAGGATTTTAATTTTTTTGTTAGAATTTTTGC

NCCR_VP2-10 (429) AACTAAAAAAAGAGCTCAAAGGATTTTAATTTTTTTGTTAGAATTTTTGC

NCCR_VP2-11 (429) AACTAAAAAAAGAGCTCAAAGGATTTTAATTTTTTTGTTAGAATTTTTGC

551 600

NCCR_VP2-01 (479) TGGACTTTTGCACAGGTGAAGACAGTGTAGACGGGAAAAAAAGACAGAGA

NCCR_VP2-02 (408) TGGACTTTTGCACAGGTGAAGACAGTGTAGACGGGAAAAAAAGACAGAGA

NCCR_VP2-05 (530) TGGACTTTTGCACAGGTGAAGACAGTGTAGACGGGAAAAAAAGACAGAGA

NCCR_VP2-06 (404) TGGACTTTTGCACAGGTGAAGACAGTGTAGACGGGAAAAAAAGACAGAGA

NCCR_VP2-07 (396) TGGACTTTTGCACAGGTGAAGACAGTGTAGACGGGAAAAAAAGACAGAGA

NCCR_VP2-08 (500) TGGACTTTTGCACAGGTGAAGACAGTGTAGACGGGAAAAAAAGACAGAGA

NCCR_VP2-04 (479) TGGACTTTTGCACAGGTGAAGACAGTGTAGACGGGAAAAAA-GACAGAGA

NCCR_VP2-03 (479) TGGACTTTTGCACAGGTGAAGACAGTGTAGACGGGAAAAAAAGACAGAGA

NCCR_VP2-09 (479) TGGACTTTTGCACAGGTGAAGACAGTGTAGACGGGAAAAAAAGACAGAGA

NCCR_VP2-10 (479) TGGACTTTTGCACAGGTGAAGACAGTGTAGACGGGAAAAAAAGACAGAGA

NCCR_VP2-11 (479) TGGACTTTTGCACAGGTGAAGACAGTGTAGACGGGAAAAAAAGACAGAGA

Agno del(51-end)

601 650

NCCR_VP2-01 (529) CACAGTGGTTTGACTCAGCAGACATACAGTGCTTTGCCTGAACCAAAAGC

NCCR_VP2-02 (458) CACAGTGGTTTGACTGAGCAGACATACAGTGCTTTGCCTGAACCAAAAGC

NCCR_VP2-05 (580) CACAGTGGTTTGACTGAGCAGACATACAGTGCTTTGCCTGAACCAAAAGC

NCCR_VP2-06 (454) CACAGTGGTTTGACTCAGCAGACATACAGTGCTTTGCCTGAACCAAAAGC

NCCR_VP2-07 (446) CACAGTGGTTTGACTCAGCAGACATACAGTGCTTTGCCTGAACCAAAAGC

NCCR_VP2-08 (550) CACAGTGGTTTGACTCAGCAGACATACAGTGCTTTGCCTGAACCAAAAGC

NCCR_VP2-04 (528) CACAGTGGTTTGACTCAGCAGACATACAGTGCTTTGCCTGAACCAAAAGC

NCCR_VP2-03 (529) CACAGTGGTTTGACTCAGCAGACATACAGTGCTTTGCCTGAACCAAAAGC

NCCR_VP2-09 (529) CACAGTGGTTTGACTCAGCAGACATACAGTGCTTTGCCTGAACCAAAAGC

NCCR_VP2-10 (529) CACAGTGGTTTGACTCAGCAGACATACAGTGCTTTGCCTGAACCAAAAGC

NCCR_VP2-11 (529) CACAGTGGTTTGACTCAGCAGACATACAGTGCTTTGCCTGAACCAAAAGC

Agno E59Q

651 700

NCCR_VP2-01 (579) TACATAGGTAAGTAATGTTTTTTTTTGTGTTTTCAGGTTCATGGGTGCCG

NCCR_VP2-02 (508) TACATAGGTAAGTAATGTTTTTTTTTGTGTTTTCAGGTTCATGGGTGCCG

NCCR_VP2-05 (630) TACATAGGTAAGTAATGTTTTTTTTTGTGTTTTCAGGTTCATGGGTGCCG NCCR_VP2-06 (504) TACATAGGTAAGTAATGTTTTTTTTTGTGTTTTCAGGTTCATGGGTGCCG

NCCR_VP2-07 (496) TACATAGGTAAGTAATGTTTTTTTTTGTGTTTTCAGGTTCATGGGTGCCG NCCR_VP2-08 (600) TACATAGGTAAGTAATGTTTTTTTTTGTGTTTTCAGGTTCATGGGTGCCG NCCR_VP2-04 (578) TACATAGGTAAGTAATGTTTTTTTTTGTGTTTTCAGGTTCATGGGTGCCG

NCCR_VP2-03 (579) TACATAGGTAAGTAATGTTTTTTTTTGTGTTTTCAGGTTCATGGGTGCCG

NCCR_VP2-09 (579) TACATAGGTAAGTAATGTTTTTTTTTGTGTTTTCAGGTTCATGGGTGCCG NCCR_VP2-10 (579) TACATAGGTAAGTAATGTTTTTTTTTGTGTTTTCAGGTTCATGGGTGCCG NCCR_VP2-11 (579) TACATAGGTAAGTAATGTTTTTTTTTGTGTTTTCAGGTTCATGGGTGCCG

Agno stop VP2

701 750

NCCR_VP2-01 (629) CACTTGCACTTTTGGGGGACCTAGTTGCTACTGTTTCTGAGGCTGCTGCT

NCCR_VP2-02 (558) CACTTGCACTTTTGGGGGACCTAGTTGCTACTGTTTCTGAGGCTGCTGCT

NCCR_VP2-05 (680) CACTTGCACTTTTGGGGGACCTAGTTGCTACTGTTTCTGAGGCTGCTGCT

NCCR_VP2-06 (554) CACTTGCACTTTTGGGGGACCTAGTTGCTACTGTTTCTGAGGCTGCTGCT

NCCR_VP2-07 (546) CACTTGCACTTTTGGGGGACCTAGTTGCTACTGTTTCTGAGGCTGCTGCT

NCCR_VP2-08 (650) CACTTGCACTTTTGGGGGACCTAGTTGCTACTGTTTCTGAGGCTGCTGCT

NCCR_VP2-04 (628) CACTTGCACTTTTGGGGGACCTAGTTGCTACTGTTTCTGAGGCTGCTGCT

NCCR_VP2-03 (629) CACTTGCACTTTTGGGGGACCTAGTTGCTACTGTTTCTGAGGCTGCTGCT

NCCR_VP2-09 (629) CACTTGCACTTTTGGGGGACCTAGTTGCTACTGTTTCTGAGGCTGCTGCT

NCCR_VP2-10 (629) CACTTGCACTTTTGGGGGACCTAGTTGCTACTGTTTCTGAGGCTGCTGCT

NCCR_VP2-11 (629) CACTTGTACTTTTGGGGGACCTAGTTGCTACTGTTTCTGAGGCTGCTGCT

VP2 A6V

751 800

NCCR_VP2-01 (679) GCCACAGGATTTTCAGTAGCTGAAATTGCTGCTGGAGAGGCTGCTGCTAC

NCCR_VP2-02 (608) GCCACAGGATTTTCAGTAGCTGAAATTGCTGCTGGAGAGGCTGCTGCTAC

NCCR_VP2-05 (730) GCCACAGGATTTTCAGTAGCTGAAATTGCTGCTGGAGAGGCTGCTGCTAC

NCCR_VP2-06 (604) GCCACAGGATTTTCAGTAGCTGAAATTGCTGCTGGAGAGGCTGCTGCTAC

NCCR_VP2-07 (596) GCCACAGGATTTTCAGTAGCTGAAATTGCTGCTGGAGAGGCTGCTGCTAC

NCCR_VP2-08 (700) GCCACAGGATTTTCAGTAGCTGAAATTGCTGCTGGAGAGGCTGCTGCTAC

NCCR_VP2-04 (678) GCCACAGGATTTTCAGTAGCTGAAATTGCTGCTGGAGAGGCTGCTGCTAC

NCCR_VP2-03 (679) GCCACAGGATTTTCAGTAGCTGAAATTGCTGCTGGAGAGGCTGCTGCTAC

NCCR_VP2-09 (679) GCCACAGGATTTTCAGTAGCTGAAATTGCTGCTGGAGAGGCTGCTGCTAC

NCCR_VP2-10 (679) GCCACAGGATTTTCAGTAGCTGAAATTGCTGCTGGAGAGGCTGCTGCTAC

NCCR_VP2-11 (679) GCCACAGGATTTTCAGTAGCTGAAATTGCTGCTGGAGAGGCTGCTGCTAC

801 850

NCCR_VP2-01 (729) TATAGAAGTTGAAATTGCATCCCTTGCTACTGTAGAGGGGATTACAAGTA

NCCR_VP2-02 (658) TATAGAAGTTGAAATTGCATCCCTTGCTACTGTAGAGCGGATTACAAGTA

NCCR_VP2-05 (780) TATAGAAGTTGAAATTGCATCCCTTGCTACTGTAGAGCGGATTACAAGTA

NCCR_VP2-06 (654) TATAGAAGTTGAAATTGCATCCCTTGCTACTGTAGAGGGGATTACAAGTA

NCCR_VP2-07 (646) TATAGAAGTTGAAATTGCATCCCTTGCTACTGTAGAGGGGATTACAAGTA

NCCR_VP2-08 (750) TATAGAAGTTGAAATTGCATCCCTTGCTACTGTAGAGGGGATTACAAGTA

NCCR_VP2-04 (728) TATAGAAGTTGAAATTGCATCCCTTGCTACTGTAGAGGGGATTACAAGTA

NCCR_VP2-03 (729) TATAGAAGTTGAAATTGCATCCCTTGCTACTGTAGAGGGGATTACAAGTA

NCCR_VP2-09 (729) TATAGAAGTTGAAATTGCATCCCTTGCTACTGTAGAGGGGATTACAAGTA

NCCR_VP2-10 (729) TATAGAAGTTGAAATTGCATCCCTTGCTACTGTAGAGGGGATTACAAGTA

NCCR_VP2-11 (729) TATAGAAGTTGAAATTGCATCCCTTGCTACTGTAGAGGGGATTACAAGTA

VP2 G50R

851 900

NCCR_VP2-01 (779) CCTCTGAGGCTATAGCTGCTATAGGCCTTACTCCTGAAACATATGCTGTA

NCCR_VP2-02 (708) CCTCTGAGGCTATAGCTGCTATAGGCCTTACTCCTGAAACATATGCTGTA

NCCR_VP2-05 (830) CCTCTGAGGCTATAGCTGCTATAGGCCTTACTCCTGAAACATATGCTGTA

NCCR_VP2-06 (704) CCTCTGAGGCTATAGCTGCTATAGGCCTTACTCCTGAAACATATGCTGTA

NCCR_VP2-07 (696) CCTCTGAGGCTATAGCTGCTATAGGCCTTACTCCTGAAACATATGCTGTA

NCCR_VP2-08 (800) CCTCTGAGGCTATAGCTGCTATAGGCCTTACTCCTGAAACATATGCTGTA

NCCR_VP2-04 (778) CCTCTGAGGCTATAGCTGCTATAGGCCTTACTCCTGAAACATATGTTGTA

NCCR_VP2-03 (779) CCTCTGAGGCTATAGCTGCTATAGGCCTTACTCCTGAAACATATGCTGTA

NCCR_VP2-09 (779) CCTCTGAGGCTATAGCTGCTATAGGCCTTACTCCTGAAACATATGCTGTA

NCCR_VP2-10 (779) CCTCTGAGGCTATAGCTGCTATAGGCCTTACTCCTGAAACATATGCTGTA

NCCR_VP2-11 (779) CCTCTGAGGCTATAGCTGCTATAGGCCTTACTCCTGAAACATATGCTGTA

VP2 A69V

901 950

NCCR_VP2-01 (829) ATAACTGGAGCTCCGGGGGCTGTAGCTGGGTTTGCTGCATTGGTTCAAAC

NCCR_VP2-02 (758) ATAACTGGAGCTCCGGGGGCTGTAGCTGGGTTTGCTGCATTGGTTCAAAC

NCCR_VP2-05 (880) ATAACTGGAGCTCCGGGGGCTGTAGCTGGGTTTGCTGCATTGGTTCAAAC

NCCR_VP2-06 (754) ATAACTGGAGCTCCGGGGGCTGTAGCTGGGTTTGCTGCATTGGTTCAAAC

NCCR_VP2-07 (746) ATAACTGGAGCTCCGGGGGCTGTAGCTGGGTTTGCTGCATTGGTTCAAAC

NCCR_VP2-08 (850) ATAACTGGAGCTCCGGGGGCTGTAGCTGGGTTTGCTGCATTGGTTCAAAC

NCCR_VP2-04 (828) ATAACTGGAGCTCCGGGGGCTGTAGCTGGGTTTGCTGCATTGGTTCAAAC

NCCR_VP2-03 (829) ATAACTGGAGCTCCGGGGGCTGTAGCTGGGTTTGCTGCATTGGTTCAAAC

NCCR_VP2-09 (829) ATAACTGGAGCTCCGGGGGCTGTAGCTGGGTTTGCTGCATTGGTTCAAAC

NCCR_VP2-10 (829) ATAACTGGAGCTCCGGGGGCTGTAGCTGGGTTTGCTGCATTGGTTCAAAC

NCCR_VP2-11 (829) ATAACTGGAGCTCCGGGGGCTGTAGCTGGGTTTGCTGCATTGGTTCAAAC

951 1000

NCCR_VP2-01 (879) TGTAACTGGTGGTAGTGCTATTGCTCAGTTGGGATATAGATTTTTTGCTG

NCCR_VP2-02 (808) TGTAACTGGTGGTAGTGCTATTGCTCAGTTGGGATATAGATTTTTTGCTG

NCCR_VP2-05 (930) TGTAACTGGTGGTAGTGCTATTGCTCAGTTGGGATATAGATTTTTTGCTG

NCCR_VP2-06 (804) TGTAACTGGTGGTAGTGCTATTGCTCAGTTGGGATATAGATTTTTTGCTG

NCCR_VP2-07 (796) TGTAACTGGTGGTAGTGCTATTGCTCAGTTGGGATATAGATTTTTTGCTG

NCCR_VP2-08 (900) TGTAACTGGTGGTAGTGCTATTGCTCAGTTGGGATATAGATTTTTTGCTG

NCCR_VP2-04 (878) TGTAACTGGTGGTAGTGCTATTGCTCAGTTGGGATATAGATTTTTTGCTG

NCCR_VP2-03 (879) TGTAACTGGTGGTAGTGCTATTGCTCAGTTGGGATATAGATTTTTTGCTG

NCCR_VP2-09 (879) TGTAACTGGTGGTAGTGCTATTGCTCAGTTGGGATATAGATTTTTTGTTG

NCCR_VP2-10 (879) TGTAACTGGTGGTAGTGCTATTGCTCAGTTGGGATATAGATTTTTTGCTG

NCCR_VP2-11 (879) TGTAACTGGTGGTAGTGCTATTGCTCAGTTGGGATATAGATTTTTTGCTG

VP2 A103V

1001 1050

NCCR_VP2-01 (929) ACTGGGATCATAAAGTTTCAACAGTTGGGCTTTTTCAGCAGCCAGCTATG

NCCR_VP2-02 (858) ACTGGGATCATAAAGTTTCAACAGTTGGGCTTTTTCAGCAGCCAGCTATG

NCCR_VP2-05 (980) ACTGGGATCATAAAGTTTCAACAGTTGGGCTTTTTCAGCAGCCAGCTATG

NCCR_VP2-06 (854) ACTGGGATCATAAAGTTTCAACAGTTGGGCTTTTTCAGCAGCCAGCTATG

NCCR_VP2-07 (846) ACTGGGATCATAAAGTTTCAACAGTTGGGCTTTTTCAGCAGCCAGCTATG

NCCR_VP2-08 (950) ACTGGGATCATAAAGTTTCAACAGTTGGGCTTTTTCAGCAGCCAGCTATG

NCCR_VP2-04 (928) ACTGGGATCATAAAGTTTCAACAGTTGGGCTTTTTCAGCAGCCAGCTATG

NCCR_VP2-03 (929) ACTGGGATCATAAAGTTTCAACAGTTGGGCTTTTTCAGCAGCCAGCTATG

NCCR_VP2-09 (929) ACTGGGATCATAAAGTTTCAACAGTTGGGCTTTTTCAGCAGCCAGCTATG

NCCR_VP2-10 (929) ACTGGGATCATAAAGTTTCAACAGTTGGGCTTTTTCAGCAGCCAGCTATG

NCCR_VP2-11 (929) ACTGGGATCATAAAGTTTCAACAGTTGGGCTTTTTCAGCAGCCAGCTATG

VP3

1051 1100

NCCR_VP2-01 (979) GCTTTACAATTATTTAATCCAGAAGACTACTATGATATATTATTTCCTGG

NCCR_VP2-02 (908) GCTTTACAATTATTTAATCCAGAAGACTACTATGATATTTTATTTCCTGG

NCCR_VP2-05 (1030) GCTTTACAATTATTTAATCCAGAAGACTACTATGATATTTTATTTCCTGG

NCCR_VP2-06 (904) GCTTTACAATTATTTAATCCAGAAGACTACTATGATATATTATTTCCTGG

NCCR_VP2-07 (896) GCTTTACAATTATTTAATCCAGAAGACTACTATGATATATTATTTCCTGG

NCCR_VP2-08 (1000) GCTTTACAATTATTTAATCCAGAAGACTACTATGATATATTATTTCCTGG

NCCR_VP2-04 (978) GCTTTACAATTATTTAATCCAGAAGACTACTATGATATATTATTTCCTGG

NCCR_VP2-03 (979) GCTTTACAATTATTTAATCCAGAAGACTACTATGATATATTATTTCCTGG

NCCR_VP2-09 (979) GCTTTACAATTATTTAATCCAGAAGACTACTATGATATATTATTTCCTGG

NCCR_VP2-10 (979) GCTTTACAATTATTTAATCCAGAAGACTACTATGATATATTATTTCCTGG

NCCR_VP2-11 (979) GCTTTACAATTATTTAATCCAGAAGACTACTATGATATATTATTTCCTGG

*

1101 1150

NCCR_VP2-01 (1029) AGTGAATGCCTTTGTTAACAATATTCACTATTTAGATCCTAGACATTGGG

NCCR_VP2-02 (958) AGTGAATGCCTTTGTTAACAATATTCACTATTTAGATCCTAGACATTGGG

NCCR_VP2-05 (1080) AGTGAATGCCTTTGTTAACAATATTCACTATTTAGATCCTAGACATTGGG

NCCR_VP2-06 (954) AGTGAATGCCTTTGTTAACAATATTCACTATTTAGATCCTAGACATTGGG

NCCR_VP2-07 (946) AGTGAATGCCTTTGTTAACAATATTCACTATTTAGATCCTAGACATTGGG

NCCR_VP2-08 (1050) AGTGAATGCCTTTGTTAACAATATTCACTATTTAGATCCTAGACATTGGG

NCCR_VP2-04 (1028) AGTGAATGCCTTTGTTAACAATATTCACTATTTAGATCCTAGACATTGGG

NCCR_VP2-03 (1029) AGTGAATGCCTTTGTTAACAATATTCACTATTTAGATCCTAGACATTGGG

NCCR_VP2-09 (1029) AGTGAATGCCTTTGTTAACAATATTCACTATTTAGATCCTAGACATTGGG

NCCR_VP2-10 (1029) AGTGAATGCCTTTGTTAACAATATTCACTATTTAGATCCTAGACATTGGG

NCCR_VP2-11 (1029) AGTGAATGCCTTTGTTAACAATATTCACTATTTAGATCCTAGACATTGGG

1151 1200

NCCR_VP2-01 (1079) GCCCGTCCTTGTTCTCCACAATCTCCCAGGCTTTTTGGAATCTTGTTAGA

NCCR_VP2-02 (1008) GCCCGTCCTTGTTCTCCACAATCTCCCAGGCTTTTTGGAATCTTGTTAGA

NCCR_VP2-05 (1130) GCCCGTCCTTGTTCTCCACAATCTCCCAGGCTTTTTGGAATCTTGTTAGA

NCCR_VP2-06 (1004) GCCCGTCCTTGTTCTCCACAATTTCCCAGGCTTTTTGGAATCTTGTTAGA

NCCR_VP2-07 (996) GCCCGTCCTTGTTCTCCACAATCTCCCAGGCTTTTTGGAATCTTGTTAGA

NCCR_VP2-08 (1100) GCCCGTCCTTGTTCTCCACAATCTCCCAGGCTTTTTGGAATCTTGTTAGA

NCCR_VP2-04 (1078) GCCCGTCCTTGTTCTCCACAATCTCCCAGGCTTTTTGGAATCTTGTTAGA

NCCR_VP2-03 (1079) GCCCGTCCTTGTTCTCCACAATCTCCCAGGCTTTTTGGAATCTTGTTAGA

NCCR_VP2-09 (1079) GCCCGTCCTTGTTCTCCACAATCTCCCAGGCTTTTTGGAATCTTGTTAGA

NCCR_VP2-10 (1079) GCCCGTCCTTGTTCTCCACAATCTCCCAGGCTTTTTGGAATCTTGTTAGA

NCCR_VP2-11 (1079) GCCCGTCCTTGTTCTCCACAATCTCCCAGGCTTTTTGGAATCTTGTTAGA

*

1201 1250

NCCR_VP2-01 (1129) GATGATTTGCCAGCCTTAACCTCTCAGGAAATTCAGAGAAGAACCCAAAA

NCCR_VP2-02 (1058) GATGATTTGCCAGCCTTAACCTCTCAGGAAATTCAGAGAAGAACCCAAAA

NCCR_VP2-05 (1180) GATGATTTGCCAGCCTTAACCTCTCAGGAAATTCAGAGAAGAACCCAAAA

NCCR_VP2-06 (1054) GATGATTTGCCAGCCTTAACCTCTCAGGAAATTCAGAGAAGAACCCAAAA

NCCR_VP2-07 (1046) GATGATTTGCCAGCCTTAACCTCTCAGGAAATTCAGAGAAGAACCCAAAA

NCCR_VP2-08 (1150) GATGATTTGCCAGCCTTAACCTCTCAGGAAATTCAGAGAAGAACCCAAAA

NCCR_VP2-04 (1128) GATGATTTGCCAGCCTTAACCTCTCAGGAAATTCAGAGAAGAACCCAAAA

NCCR_VP2-03 (1129) GATGATTTGCCAGCCTTAACCTCTCAGGAAATTCAGAGAAGAACCCAAAA

NCCR_VP2-09 (1129) GATGATTTGCCAGCCTTAACCTCTCAGGAAATTCAGAGAAGAACCCAAAA

NCCR_VP2-10 (1129) GATGATTTGCCAGCCTTAACCTCTCAGGAAATTCAGAGAAGAACCCAAAA

NCCR_VP2-11 (1129) GATGATTTGCCAGCCTTAACCTCTCAGGAAATTCAGAGAAGAACCCAAAA

1251 1300

NCCR_VP2-01 (1179) ACTATTTGTTGAAAATTTAGCAAGGTTTTTGGAAGAAACTACTTGGGCAA

NCCR_VP2-02 (1108) GCTATTTGTTGAAAATTTAGCAAGGTTTTTGGAAGAAACTACTTGGGCAA

NCCR_VP2-05 (1230) GCTATTTGTTGAAAATTTAGCAAGGTTTTTGGAAGAAACTACTTGGGCAA

NCCR_VP2-06 (1104) ACTATTTGTTGAAAATTTAGCAAGGTTTTTGGAAGAAACTACTTGGGCAA

NCCR_VP2-07 (1096) ACTATTTGTTGAAAATTTAGCAAGGTTTTTGGAAGAAACTACTTGGGCAA

NCCR_VP2-08 (1200) ACTATTTGTTGAAAATTTAGCAAGGTTTTTGGAAGAAACTACTTGGGCAA

NCCR_VP2-04 (1178) ACTATTTGTTGAAAATTTAGCAAGGTTTTTGGAAGAAACTACTTGGGCAA

NCCR_VP2-03 (1179) ACTATTTGTTGAAAATTTAGCAAGGTTTTTGGAAGAAACTACTTGGGCAA

NCCR_VP2-09 (1179) ACTATTTGTTGAAAATTTAGCAAGGTTTTTGGAAGAAACTACTTGGGCAA

NCCR_VP2-10 (1179) ACTATTTGTTGAAAATTTAGCAAGGTTTTTGGAAGAAACTACTTGGGCAA

NCCR_VP2-11 (1179) ACTATTTGTTGAAAATTTAGCAAGGTTTTTGGAAGAAACTACTTGGGCAA

*

1301 1350

NCCR_VP2-01 (1229) TAGTTAATTCACCAGCTAACTTATATAATTATATTTCAGACTATTATTCT

NCCR_VP2-02 (1158) TAGTTAATTCACCAGCTAACTTATATAATTATATTTCAGACTATTATTCT

NCCR_VP2-05 (1280) TAGTTAATTCACCAGCTAACTTATATAATTATATTTCAGACTATTATTCT

NCCR_VP2-06 (1154) TAGTTAATTCACCAGCTAACTTATATAATTATATTTCAGACTATTATTCT

NCCR_VP2-07 (1146) TAGTTAATTCACCAGCTAACTTATATAATTATATTTCAGACTATTATTCT

NCCR_VP2-08 (1250) TAGTTAATTCACCAGCTAACTTATATAATTATATTTCAGACTATTATTCT

NCCR_VP2-04 (1228) TAGTTAATTCACCAGCTAACTTATATAATTATATTTCAGACTATTATTCT

NCCR_VP2-03 (1229) TAGTTAATTCACCAGCTAACTTATATAATTATATTTCAGACTATTATTCT

NCCR_VP2-09 (1229) TAGTTAATTCACCAGCTAACTTATATAATTATATTTCAGACTATTATTCT

NCCR_VP2-10 (1229) TAGTTAATTCACCAGCTAACTTATATAATTATATTTCAGACTATTATTCT

NCCR_VP2-11 (1229) TAGTTAATTCACCAGCTAACTTATATAATTATATTTCAGACTATTATTCT

1351 1400

NCCR_VP2-01 (1279) AGATTGTCTCCAGTTAGGCCCTCTATGGTAAGGCAAGTTGCCCAAAGGGA

NCCR_VP2-02 (1208) AGATTGTCTCCAGTTAGGCCCTCTATGGTAAGGCAAGTTGCCCAAAGGGA

NCCR_VP2-05 (1330) AGATTGTCTCCAGTTAGGCCCTCTATGGTAAGGCAAGTTGCCCAAAGGGA

NCCR_VP2-06 (1204) AGATTGTCTCCAGTTAGGCCCTCTATGGTAAGGCAAGTTGCCCAAAGGGA

NCCR_VP2-07 (1196) AGATTGTCTCCAGTTAGGCCCTCTATGGTAAGGCAAGTTGCCCAAAGGGA

NCCR_VP2-08 (1300) AGATTGTCTCCAGTTAGGCCCTCTATGGTAAGGCAAGTTGCCCAAAGGGA

NCCR_VP2-04 (1278) AGATTGTCTCCAGTTAGGCCCTCTATGGTAAGGCAAGTTGCCCAAAGGGA

NCCR_VP2-03 (1279) AGATTGTCTCCAGTTAGGCCCTCTATGGTAAGGCAAGTTGCCCAAAGGGA

NCCR_VP2-09 (1279) AGATTGTCTCCAGTTAGGCCCTCTATGGTAAGGCAAGTTGCCCAAAGGGA

NCCR_VP2-10 (1279) AGATTGTCTCCAGTTAGGCCCTCTATGGTAAGGCAAGTTACCCAAAGGGA

NCCR_VP2-11 (1279) AGATTGTCTCCAGTTAGGCCCTCTATGGTAAGGCAAGTTGCCCAAAGGGA

VP2 A234T

1401 1450

NCCR_VP2-01 (1329) GGGAACCTATATTTCTTTTGGCCACTCATACACCCAAAGTATAGATGATG

NCCR_VP2-02 (1258) GGGAACCTATATTTCTTTTGGCCACTCATACACCCAAAGTATAGATGATG

NCCR_VP2-05 (1380) GGGAACCTATATTTCTTTTGGCCACTCATACACCCAAAGTATAGATGATG

NCCR_VP2-06 (1254) GGGAACCTATATTTCTTTTGGCCACTCATACACCCAAAGTATAGATGATG

NCCR_VP2-07 (1246) GGGAACCTATATTTCTTTTGGCCACTCATACACCCAAAGTATAGATGATG

NCCR_VP2-08 (1350) GGGAACCTATATTTCTTTTGGCCACTCATACACCCAAAGTATAGATGATG

NCCR_VP2-04 (1328) GGGAACCTATATTTCTTTTGGCCACTCATACACCCAAAGTATAGATGATG

NCCR_VP2-03 (1329) GGGAACCTATATTTCTTTTGGCCACTCATACACCCAAAGTATAGATGATG

NCCR_VP2-09 (1329) GGGAACCTATATTTCTTTTGGCCACTCATACACCCAAAGTATAGATGATG

NCCR_VP2-10 (1329) GGGAACCTATATTTCTTTTGGCCACTCATACACCCAAAGTATAGATGATG

NCCR_VP2-11 (1329) GGGAACCTATATTTCTTTTGGCCACTCATACACCCAAAGTATAGATGATG

1451 1500

NCCR_VP2-01 (1379) CAGACAGCATTCAAGAAGTTACCCAAAGGCTAGATTTAAAAACCCCAAAT

NCCR_VP2-02 (1308) CAGACAGCATTCAAGAAGTTACCCAAAGGCTAGATTTAAAAACCCCAAAT

NCCR_VP2-05 (1430) CAGACAGCATTCAAGAAGTTACCCAAAGGCTAGATTTAAAAACCCCAAAT

NCCR_VP2-06 (1304) CAGACAGCATTCAAGAAGTTACCCAAAGGCTAGATTTAAAAACCCCAAAT

NCCR_VP2-07 (1296) CAGACAGCATTCAAGAAGTTACCCAAAGGCTAGATTTAAAAACCCCAAAT

NCCR_VP2-08 (1400) CAGACAGCATTCAAGAAGTTACCCAAAGGCTAGATTTAAAAACCCCAAAT

NCCR_VP2-04 (1378) CAGACAGCATTCAAGAAGTTACCCAAAGGCTAGATTTAAAAACCCCAAAT

NCCR_VP2-03 (1379) CAGACAGCATTCAAGAAGTTACCCAAAGGCTAGATTTAAAAACCCCAAAT

NCCR_VP2-09 (1379) CAGACAGCATTCAAGAAGTTACCCAAAGGCTAGATTTAAAAACCCCAAAT

NCCR_VP2-10 (1379) CAGACAGCATTCAAGAAGTTACCCAAAGGCTAGATTTAAAAACCCCAAAT

NCCR_VP2-11 (1379) CAGACAGCATTCAAGAAGTTACCCAAAGGCTAGATTTAAAAACCCCAAAT

1501 1550

NCCR_VP2-01 (1429) GTGCAATCTGGTGAATTTATAGAAAGAAGTATTGCACCAGGAGGTGCAAA

NCCR_VP2-02 (1358) GTGCAATCTGGTGAATTTATAGAAAGAAGTATTGCACCAGGAGGTGCAAA

NCCR_VP2-05 (1480) GTGCAATCTGGTGAATTTATAGAAAGAAGTATTGCACCAGGAGGTGCAAA

NCCR_VP2-06 (1354) GTGCAATCTGGTGAATTTATAGAAAGAAGTATTGC---------------

NCCR_VP2-07 (1346) GTGCAATCTGGTGAATTTATAGAAAGAAGTATTGCACCAGGAGGTGCAAA

NCCR_VP2-08 (1450) GTGCAATCTGGTGAATTTATAGAAAGAAGTATTGCACCAGGAGGTGCAAA

NCCR_VP2-04 (1428) GTGCAATCTGGTGAATTTATAGAAAGAAGTATTGCACCAGGAGGTGCAAA

NCCR_VP2-03 (1429) GTGCAATCTGGTGAATTTATAGAAAGAAGTATTGCACCAGGAGGTGCAAA

NCCR_VP2-09 (1429) GTGCAATCTGGTGAATTTATAGAAAGAAGTATTGCACCAGGAGGTGCAAA

NCCR_VP2-10 (1429) GTGCAATCTGGTGAATTTATAGAAAGAAGTATTGCACCAGGAGGTGCAAA

NCCR_VP2-11 (1429) GTGCAATCTGGTGAATTTATAGAAAGAAGTATTGCACCAGGAGGTGCAAA

VP2 del(283-end)

1551 1600

NCCR_VP2-01 (1479) TCAAAGATCTGCTCCTCAATGGATGTTGCCTTTACTTTTAGGGTTGTACG

NCCR_VP2-02 (1408) TCAAAGATCTGCTCCTCAATGGATGTTGCCTTTACTTTTAGGGTTGTACG

NCCR_VP2-05 (1530) TCAAAGATCTGCTCCTCAATGGATGTTGCCTTTACTTTTAGGGTTGTACG

NCCR_VP2-06 (1389) --------------------------------------------------

NCCR_VP2-07 (1396) TCAAAGATCTGCTCCTCAATGGATGTTGCCTTTACTTTTAGGGTTGTACG

NCCR_VP2-08 (1500) TCAAAGATCTGCTCCTCAATGGATGTTGCCTTTACTTTTAGGGTTGTACG

NCCR_VP2-04 (1478) TCAAAGATCTGCTCCTCAATGGATGTTGCCTTTACTTTTAGGGTTGTACG

NCCR_VP2-03 (1479) TCAAAGATCTGCTCCTCAATGGATGTTGCCTTTACTTTTAGGGTTGTACG

NCCR_VP2-09 (1479) TCAAAGATCTGCTCCTCAATGGATGTTGCCTTTACTTTTAGGGTTGTACG

NCCR_VP2-10 (1479) TCAAAGATCTGCTCCTCAATGGATGTTGCCTTTACTTTTAGGGTTGTACG

NCCR_VP2-11 (1479) TCAAAGATCTGCTCCTCAATGGATGTTGCCTTTACTTTTAGGGTTGTACG

1601 1650

NCCR_VP2-01 (1529) GGACTGTAACACCTGCTCTTGAAGCATATGAAGATGGCCCCAACAAAAAG

NCCR_VP2-02 (1458) GGACTGTAACACCTGCTCTTGAAGCATATGAAGATGGCCCCAACAAAAAG

NCCR_VP2-05 (1580) GGACTGTAACACCTGCTCTTGAAGCATATGAAGATGGCCCCAACAAAAAG

NCCR_VP2-06 (1389) --------------------------------------------------

NCCR_VP2-07 (1446) GGACTGTAACACCTGCTCTTGAAGCATATGAAGATGGCCCCAACAAAAAG

NCCR_VP2-08 (1550) GGACTGTAACACCTGCTCTTGAAGCATATGAAGATGGCCCCAACAAAAAG

NCCR_VP2-04 (1528) GGACTGTAACACCTGCTCTTGAAGCATATGAAGATGGCCCCAACAAAAAG

NCCR_VP2-03 (1529) GGACTGTAACACCTGCTCTTGAAGCATATGAAGATGGCCCCAACAAAAAG

NCCR_VP2-09 (1529) GGACTGTAACACCTGCTCTTGAAGCATATGAAGATGGCCCCAACAAAAAG

NCCR_VP2-10 (1529) GGACTGTAACACCTGCTCTTGAAGCATATGAAGATGGCCCCAACAAAAAG

NCCR_VP2-11 (1529) GGACTGTAACACCTGCTCTTGAAGCATATGAAGATGGCCCCAACAAAAAG

VP1

1651 1700

NCCR_VP2-01 (1579) AAAAGCAGAAAGGAAGGACCCCGTGCAAGTTCCAAAACTTCTTATAAGAG

NCCR_VP2-02 (1508) AAAAGGAGAAAGGAAGGACCCCGTGCAAGTTCCAAAACTTCTTATAAGAG

NCCR_VP2-05 (1630) AAAAGCAGAAAGGAAGGACCCCGTGCAAGTTCCAAAACTTCTTATAAGAG

NCCR_VP2-06 (1389) --------------------------------------------------

NCCR_VP2-07 (1496) AAAAGCAGAAAGGAAGGACCCCGTGCAAGTTCCAAAACTTCTTATAAGAG

NCCR_VP2-08 (1600) AAAAGCAGAAAGGAAGGACCCCGTGCAAGTTCCAAAACTTCTTATAAGAG

NCCR_VP2-04 (1578) AAAAGCAGAAAGGAAGGACCCCGTGCAAGTTCCAAAACTTCTTATAAGAG

NCCR_VP2-03 (1579) AAAAGCAGAAAGGAAGGACCCCGTGCAAGTTCCAAAACTTCTTATAAGAG

NCCR_VP2-09 (1579) AAAAGCAGAAAGGAAGGACCCCGTGCAAGTTCCAAAACTTCTTATAAGAG

NCCR_VP2-10 (1579) AAAAGCAGAAAGGAAGGACCCCGTGCAAGTTCCAAAACTTCTTATAAGAG

NCCR_VP2-11 (1579) AAAAGCAGAAAGGAAGGACCCCGTGCAAGTTCCAAAACTTCTTATAAGAG

VP2 R322S

1701 1750

NCCR_VP2-01 (1629) GAGGAGTAGAAGTTCTAGAAGTTAAAACTGGGGTTGACTCAATTACAGAG

NCCR_VP2-02 (1558) GAGGAGTAGAAGTTCTAGAAGTTAAAACTGGGGTTGACTCAATTACAGAG

NCCR_VP2-05 (1680) GAGGAGTAGAAGTTCTAGAAGTTAAAACTGGGGTTGACTCAATTACAGAG

NCCR_VP2-06 (1389) --------------CTAGAAGTTAAAACTGGGGTTGACTCAATTACAGAG

NCCR_VP2-07 (1546) GAGGAGTAGAAGTTCTAGAAGTTAAAACTGGGGTTGACTCAATTACAGAG

NCCR_VP2-08 (1650) GAGGAGTAGAAGTTCTAGAAGTTAAAACTGGGGTTGACTCAATTACAGAG

NCCR_VP2-04 (1628) GAGGAGTAGAAGTTCTAGAAGTTAAAACTGGGGTTGACTCAATTACAGAG

NCCR_VP2-03 (1629) GAGGAGTAGAAGTTCTAGAAGTTAAAACTGGGGTTGACTCAATTACAGAG

NCCR_VP2-09 (1629) GAGGAGTAGAAGTTCTAGAAGTTAAAACTGGGGTTGACTCAATTACAGAG

NCCR_VP2-10 (1629) GAGGAGTAGAAGTTCTAGAAGTTAAAACTGGGGTTGACTCAATTACAGAG

NCCR_VP2-11 (1629) GAGGAGTAGAAGTTCTAGAAGTTAAAACTGGGGTTGACTCAATTACAGAG

VP2 del(283-end) stop stop

1751 1800

NCCR_VP2-01 (1679) GTAGAATGCTTTTTAACTCCAGAAATGGGTGACCCAGATGAGCATCTTAG

NCCR_VP2-02 (1608) GTAGAATGCTTTTTAACTCCAGAAATGGGTGACCCAGATGAGCATCTTAG

NCCR_VP2-05 (1730) GTAGAATGCTTTTTAACTCCAGAAATGGGTGACCCAGATGAGCATCTTAG

NCCR_VP2-06 (1425) GTAGAATGCTTTTTAACTCCAGAAATGGGTGACCCAGATGAGCATCTTAG

NCCR_VP2-07 (1596) GTAGAATGCTTTTTAACTCCAGAAATGGGTGACCCAGATGAGCATCTTAG

NCCR_VP2-08 (1700) GTAGAATGCTTTTTAACTCCAGAAATGGGTGACCCAGATGAGCATCTTAG

NCCR_VP2-04 (1678) GTAGAATGCTTTTTAACTCCAGAAATGGGTGACCCAGATGAGCATCTTAG

NCCR_VP2-03 (1679) GTAGAATGCTTTTTAACTCCAGAAATGGGTGACCCAGATGAGCATCTTAG

NCCR_VP2-09 (1679) GTAGAATGCTTTTTAACTCCAGAAATGGGTGACCCAGATGAGCATCTTAG

NCCR_VP2-10 (1679) GTAGAATGCTTTTTAACTCCAGAAATGGGTGACCCAGATGAGCATCTTAG

NCCR_VP2-11 (1679) GTAGAATGCTTTTTAACTCCAGAAATGGGTGACCCAGATGAGCATCTTAG

1801 1850

NCCR_VP2-01 (1729) GGGTTTTAGTAAGTCAATATCTATATCAGATACATTTGAAAGTGACTCCC

NCCR_VP2-02 (1658) GGGTTTTAGTAAGTCAATATCTATATCAGATACATTTGAAAGTGACTCCC

NCCR_VP2-05 (1780) GGGTTTTAGTAAGTCAATATCTATATCAGATACATTTGAAAGTGACTCCC

NCCR_VP2-06 (1475) GGGTTTTAGTAAGTCAATATCTATATCAGATACATTTGAAAGTGACTCCC

NCCR_VP2-07 (1646) GGGTTTTAGTAAGTCAATATCTATATCAGATACATTTGAAAGTGACTCCC

NCCR_VP2-08 (1750) GGGTTTTAGTAAGTCAATATCTATATCAGATACATTTGAAAGTGACTCCC

NCCR_VP2-04 (1728) GGGTTTTAGTAAGTCAATATCTATATCAGATACATTTGAAAGTGACTCCC

NCCR_VP2-03 (1729) GGGTTTTAGTAAGTCAATATCTATATCAGATACATTTGAAAGTGACTCCC

NCCR_VP2-09 (1729) GGGTTTTAGTAAGTCAATATCTATATCAGATACATTTGAAAGTGACTCCC

NCCR_VP2-10 (1729) GGGTTTTAGTAAGTCAATATCTATATCAGATACATTTGAAAGTGACTCCC

NCCR_VP2-11 (1729) GGGTTTTAGTAAGTCAATATCTATATCAGATACATTTGAAAGTGACTCCC

1851 1900

NCCR_VP2-01 (1779) CAAATAAGGACATGCTTCCTTGTTACAGTGTGGCCAG*AAGGGCGAATTC*-

NCCR_VP2-02 (1708) CAAGTAAGGACATGCTTCCTTGTTACAGTGTGGCCAG*AAGGGCGAATTC*-

NCCR_VP2-05 (1830) CAAATAAGGACATGCTTCCTTGTTACAGTGTGGCCAG*AAGGGCGAATTC*-

NCCR_VP2-06 (1525) CAAGTAAGGACATGCTTCCTTGTTACAGTGTGGCCAG*AAGGGCGAATTC*-

NCCR_VP2-07 (1696) CAAATAAGGACATGCTTCCTTGTTACAGTGTGGCCAG*AAGGGCGAATTG-*

NCCR_VP2-08 (1800) CAAATAAGGACATGCTTCCTTGTTACAGTGTGGCCAG*AAGGGCGAATTC*-

NCCR_VP2-04 (1778) CAAGTAAGGACATGCTTCCTTGTTACAGTGTGGCCAG*AAGGGCGAATTC*-

NCCR_VP2-03 (1779) CAAATAAGGACATGCTTCCTTGTTACAGTGTGGCCAC*AAGGGCGAATTC*-

NCCR_VP2-09 (1779) CAAATAAGGACATGCTTCCTTGTTACAGTGTGGCCAG*AAGGGCGAATTC*-

NCCR_VP2-10 (1779) CAAATAAGGACATGCTTCCTTGTTACAGTGTGGCCCG*AAGGGCGAATTC*-

NCCR_VP2-11 (1779) CAAATAAGGACATGCTTCCTTGTTACAGTGTGGC--G*AAGGGCGAATTC*-

**

Efalizumab/PML brain DNA “VP1” sequences

Nucleotide 1 50

VP1-01 (1) *GAATTCGCCCTT*GCAGCCAGCTATGGCTTTACAATTATTTAATCCAGAAG

VP1-02 (1) *GAATTCGCCCTT*GCAGCCAGCTATGGCTTTACAATTATTTAATCCAGAAG

VP1-03 (1) *GAATTCGCCCTT*GCAGCCAGCTATGGCTTTACAATTATTTAATCCAGAAG

VP3

51 100

VP1-01 (51) ACTACTATGATATATTATTTCCTGGAGTGAATGCCTTTGTTAACAATATT

VP1-02 (51) ACTACTATGATATTTTATTTCCTGGAGTGAATGCCTTTGTTAACAATATT

VP1-03 (51) ACTACTATGATATTTTATTTCCTGGAGTGAATGCCTTTGTTAACAATATT

*

101 150

VP1-01 (101) CACTATTTAGATCCTAGACATTGGGGCCCGTCCTTGTTCTCCACAATCTC

VP1-02 (101) CACTATTTAGATCCTAGACATTGGGGCCCGTCCTTGTTCTCCACAATCTC

VP1-03 (101) CACTATTTAGATCCTAGACATTGGGGCCCGTCCTTGTTCTCCACAATCTC

151 200

VP1-01 (151) CCAGGCTTTTTGGAATCTTGTTAGAGATGATTTGTCAGCCTTAACCTCTC

VP1-02 (151) CCAGGCTTTTTGGAATCTTGTTAGAGATGATTTGCCAGCCTTAACCTCTC

VP1-03 (151) CCAGGCTTTTTGGAATCTTGTTAGAGATGATTTGCCAGCCTTAACCTCTC

VP2 P174S

VP3 P55S

201 250

VP1-01 (201) AGGAAATTCAGAGAAGAACCCAAAAACTATTTGTTGAAAATTTAGCAAGG

VP1-02 (201) AGGAAATTCAGAGAAGAACCCAAAAGCTATTTGTTGAAAATTTAGCAAGG

VP1-03 (201) AGGAAATTCAGAGAAGAACCCAAAAGCTATTTGTTGAAAATTTAGCAAGG

*

251 300

VP1-01 (251) TTTTTGGAAGAAACTACTTGGGCAATAGTTAATTCACCAGCTAACTTATA

VP1-02 (251) TTTTTGGAAGAAACTACTTGGGCAATAGTTAATTCACCAGCTAACTTATA

VP1-03 (251) TTTTTGGAAGAAACTACTTGGGCAATAGTTAATTCACCAGCTAACTTATA

301 350

VP1-01 (301) TAATTATATTTCAGACTATTATTCTAGATTGTCTCCAGTTAGGCCCTCTA

VP1-02 (301) TAATTATATTTCAGACTATTATTCTAGATTGTCTCCAGTTAGGCCCTCTA

VP1-03 (301) TAATTATATTTCAGACTATTATTCTAGATTGTCTCCAGTTAGGCCCTCTA

351 400

VP1-01 (351) TGGTAAGGCAAGTTGCCCAAAGGGAGGGAACCTATATTTCTTTTGGCCAC

VP1-02 (351) TGGTAAGGCAAGTTGCCCAAAGGGAGGGAACCTATATTTCTTTTGGCCAC

VP1-03 (351) TGGTAAGGCAAGTTGCCCAAAGGGAGGGAACCTATATTTCTTTTGGCCAC

401 450

VP1-01 (401) TCATACACCCAAAGTATAGATGATGCAGACAGCATTCAAGAAGTTACCCA

VP1-02 (401) TCATACACCCAAAGTATAGATGATGCAGACAGCATTCAAGAAGTTACCCA

VP1-03 (401) TCATACACCCAAAGTATAGATGATGCAGACAGCATTCAAGAAGTTACCCA

451 500

VP1-01 (451) AAGGCTAGATTTAAAAACCCCAAATGTGCAATCTGGTGAATTTATAGAAA

VP1-02 (451) AAGGCTAGATTTAAAAACCCCAAATGTGCAATCTGGTGAATTTATAGAAA

VP1-03 (451) AAGGCTAGATTTAAAAACCCCAAATGTGCAATCTGGTGAATTTATAGAAA

501 550

VP1-01 (501) GAAGTATTGCACCAGGAGGTGCAAATCAAAGATCTGCTCCTCAATGGATG

VP1-02 (501) GAAGTATTGCACCAGGAGGTGCAAATCAAAGATCTGCTCCTCAATGGATG

VP1-03 (501) GAAGTATTGCACCAGGAGGTGCAAATCAAAGATCTGCTCCTCAATGGATG

551 600

VP1-01 (551) TTGCCTTTACTTTTAGGGTTGTACGGGACTGTAACACCTGCTCTTGAAGC

VP1-02 (551) TTGCCTTTACTTTTAGGGTTGTACGGGACTGTAACACCTGCTCTTGAAGC

VP1-03 (551) TTGCCTTTACTTTTAGGGTTGTACGGGACTGTAACACCTGCTCTTGAAGC

601 650

VP1-01 (601) ATATGAAGATGGCCCCAACAAAAAGAAAAGCAGAAAGGAAGGACCCCGTG

VP1-02 (601) ATATGAAGATGGCCCCAACAAAAAGAAAAGGAGAAAGGAAAGACCCCGTG

VP1-03 (601) ATATGAAGATGGCCCCAACAAAAAGAAAAGGAGAAAGGAAGGACCCCGTG

VP1 VP1 G8A *

VP2 R207G

651 700

VP1-01 (651) CAAGTTCCAAAACTTCTTATAAGAGGAGGAGTAGAAGTTCTAGAAGTTAA

VP1-02 (651) CAAGTTCCAAAACTTCTTATAAGAGGAGGAGTAGAAGTTCTAGAAGTTAA

VP1-03 (651) CAAGTTCCAAAACTTCTTATAAGAGGAGGAGTAGAAGTTCTAGAAGTTAA

VP2 stop

701 750

VP1-01 (701) AACTGGGGTTGACTCAATTACAGAGGTAGAATGCTTTTTAACTCCAGAAA

VP1-02 (701) AACTGGGGTTGACTCAATTACAGAGGTAGAATGCTTTTTAACTCCAGAAA

VP1-03 (701) AACTGGGGTTGACTCAATTACAGAGGTAGAATGCTTTTTAACTCCAGAAA

751 800

VP1-01 (751) TGGGTGACCCAGATGAGCATCTTAGGGGTTTTAGTAAGTCAATATCTATA

VP1-02 (751) TGGGTGACCCAGATGAGCATCTTAGGGGTTTTAGTAAGTCAATATCTATA

VP1-03 (751) TGGGTGACCCAGATGAGCATCTTAGGGGTTTTAGTAAGTCAATATCTATA

801 850

VP1-01 (801) TCAGATACATTTGAAAGTGACTCCCCAAATAAGGACATGCTTCCTTGTTA

VP1-02 (801) TCAGATACATTTGAAAGTGACTCCCCAAGTAAGGACATGCTTCCTTGTTA

VP1-03 (801) TCAGATACATTTGAAAGTGACTCCCCAAGTAAGGACATGCTTCCTTGTTA

VP1 S74N

851 900

VP1-01 (851) CAGTGTGGCCAGAATTCCACTACCCAATCTAAATGAGGATCTAACCTGTG

VP1-02 (851) CAGTGTGGCCAGAATTCCACTACCCAATCTAAATGAGGATCTAACCTGTG

VP1-03 (851) CAGTGTGGCCAGAATTCCACTACCCAATCTAAATGAGGATCTAACCTGTG

901 950

VP1-01 (901) GAAATATACTCATGTGGGAGGCTGTGACCTTAAAAACTGAGGTTATAGGG

VP1-02 (901) GAAATATACTCATGTGGGAGGCTGTGACCTTAAAAACTGAGGTTATAGGG

VP1-03 (901) GAAATATACTCATGTGGGAGGCTGTGACCTTAAAAACTGAGGTTATAGGG

951 1000

VP1-01 (951) GTGACAAGTTTGATGAATGTGCACTCTAATGGGCAAGCAACTCATGACAA

VP1-02 (951) GTGACAAGTTTGATGAATGTGCACTCTAATGGTCAAGCAGCTCATGACAA

VP1-03 (951) GTGACAAGTTTGATGAATGTGCACTCTAATGGTCAAGCAGCTCATGACAA

* VP1 A128T

1001 1050

VP1-01 (1001) TGGTGCAGGGAAGCCAGTGCAGGGCACCAGCTTTCATTTTTTTTCTGTTG

VP1-02 (1001) TGGTGCAGGGAAGCCAGTGCAGGGCACCAGCTTTCATTTTTTTTCTGTTG

VP1-03 (1001) TGGTGCAGGGAAGCCAGTGCAGGGCACCAGCTTTCATTTTTTTTCTGTTG

1051 1100

VP1-01 (1051) GGGGGGAGGCTTTAGAATTACAGGGGGTGGTTTTTAATTACAGAACAAAG

VP1-02 (1051) GGGGGGAGGCTTTAGAATTACAGGGGGTGGTTTTTAATTACAGAACAAAG

VP1-03 (1051) GGGGGGAGGCTTTAGAATTACAGGGGGTGGTTTTTAATTACAGAACAAAG

1101 1150

VP1-01 (1101) TACCCAGATGGAACAATTTTTCCAAAGAATGCCACAGTGCAATCTCAAGT

VP1-02 (1101) TACCCAGATGGAACAATTTTTCCAAAGAATGCTACAGTGCAATCTCAAGT

VP1-03 (1101) TACCCAGATGGAACAATTTTTCCAAAGAATGCTACAGTGCAATCTCAAGT

*

1151 1200

VP1-01 (1151) CATGAACACAGAGCACAAGGCGTACCTAGATAAGAACAAAGCATATCCTG

VP1-02 (1151) CATGAACACAGAGCACAAGGCGTACCTAGATAAGAACAAAGCATATCCTG

VP1-03 (1151) CATGAACACAGAGCACAAGGCGTACCTAGATAAGAACAAAGCATATCCTG

1201 1250

VP1-01 (1201) TTGAATGTTGGGTTCCTGATCCCACCAGAAATGAAAACACAAGATATTTT

VP1-02 (1201) TTGAATGTTGGGTTCCTGATCCCACCAGAAATGAAAACACAAGATATTTT

VP1-03 (1201) TTGAATGTTGGGTTCCTGATCCCACCAGAAATGAAAACACAAGATATTTT

1251 1300

VP1-01 (1251) GGGACACTAACAGGAGGAGAAAATGTTCCTCCAGTTCTTCATATAACAAA

VP1-02 (1251) GGGACACTAACAGGAGGAGAAAATGTTCCTCCAGTTCTTCATATAACAAA

VP1-03 (1251) GGGACACTAACAGGAGGAGAAAATGTTCCTCCAGTTCTTCATATAACAAA

1301 1350

VP1-01 (1301) CACTGCCACAACAGTGTTGCTTGATGAATTTGGTGTTGGGCCACTTTGCA

VP1-02 (1301) CACTGCCACAACAGTGTTGCTTGATGAATTTGGTGTTGGGCCACTTTGCA

VP1-03 (1301) CACTGCCACAACAGTGTTGCTTGATGAATTTGGTGTTGGGCCACTTTGCA

1351 1400

VP1-01 (1351) AAGGTGACAACTTATACTTGTCAGCTGTTGATGTTTGTGGCATGTTTACT

VP1-02 (1351) AAGGTGACAACTTATACTTGTCAGCTGTTGATGTTTGTGGCATGTTTACT

VP1-03 (1351) AAGGTGACAACTTATACTTGTCAGCTGTTGATGTTTGTGGCATGTTTACT

1401 1450

VP1-01 (1401) AACAGGTCTGGTTTCCAGCAGTGGAGAGGACTCTCCAGATATTTTAAGGT

VP1-02 (1401) AACAGGTCTGGTTCCCAGCAGTGGAGAGGACTCTCCAGATATTTTAAGGT

VP1-03 (1401) AACAGGTCTGGTTTCCAGCAGTGGAGAGGACTCTCCAGATATTTTAAGGT

VP1 S269F

1451 1500

VP1-01 (1451) TCAGCTAAGGAAAAGGAGGGTGAAAAACCCCTACCCAATTTCTTTCCTTC

VP1-02 (1451) TCAGCTAAGGAAAAGGAGGGTTAAAAACCCCTACCCAATTTCTTTCCTTC

VP1-03 (1451) TCAGCTAAGGAAAAGGAGGGTGAAAAACCCCTACCCAATTTCTTTCCTTC

*

1501 1550

VP1-01 (1501) TTACTGATTTAATTAACAGAAGGACTCCTAGAGTTGATGGGCAGCCTATG

VP1-02 (1501) TTACTGATTTAATTAACAGAAGGACTCCTAGAGTTGATGGGCAGCCTATG

VP1-03 (1501) TTACTGATTTAATTAACAGAAGGACTCCTAGAGTTGATGGGCAGCCTATG

1551 1600

VP1-01 (1551) TATGGCATGGATGCTCAAGTAGAGGAGGTTAGAGTTTTTGAGGGAACAGA

VP1-02 (1551) TATGGCATGGATGCTCAAGTAGAGGAGGTTAGAGTTTTTGAGGGAACAGA

VP1-03 (1551) TATGGCATGGATGCTCAAGTAGAGGAGGTTAGAGTTTTTGAGGGAACAGA

1601 1650

VP1-01 (1601) GGAGCTTCCAGGGGACCCAGACATGATGAGATACGTTGACAGATATGGAC

VP1-02 (1601) GGAGCTTCCAGGGGACCCAGACATGATGAGATACGTTGACAGATATGGAC

VP1-03 (1601) GGAGCTTCCAGGGGACCCAGACATGATGAGATACGTTGACAGATATGGAC

1651 1700

VP1-01 (1651) AGTTGCAGACAAAAATGCTGTAATCAAAAGCCTTTATTGTAATATGCAGT

VP1-02 (1651) AGTTGCAGACAAAAATGCTGTAATCAAAAGCCTTTATTGTAATATGCAGT

VP1-03 (1651) AGTTGCAGACAAAAATGCTGTAATCAAAAGCCTTTATTGTAATATGCAGT

VP1 stop

1701 1750

VP1-01 (1701) ACATTTTAATAAAGTATTACCAGCTTTACTTGACATTTGCAGTTATTTTG

VP1-02 (1701) ACATTTTAATAAAGTATTACCAGCTTTACTTGACATTTGCAGTTATTTTG

VP1-03 (1701) ACATTTTAATAAAGTATTACCAGCTTTACTTGACATTTGCAGTTATTTTG

1751 1800

VP1-01 (1751) GGGGAGGGGTCTTTGGTTTTTTGAAACATTGAAAGCCTTTACAGATGTGA

VP1-02 (1751) GGGGAGGGGTCTTTGGTTTTTTGAAACATTGAAAGCCTTTACAGATGTGA

VP1-03 (1751) GGGGAGGGGTCTTTGGTTTTTTGAAACATTGAAAGCCTTTACAGATGTGA

1801 1850

VP1-01 (1801) AAGGTGCAGTTTTCCTGTGTGTCTGCACCAGAGGCTTCTGAGACCTGGGA

VP1-02 (1801) AAGGTGCAGTTTTCCTGTGTGTCTGCACCAGAGGCTTCTGAGACCGGGGA

VP1-03 (1801) AAGGTGCAGTTTTCCTGTGTGTCTGCACCAGAGGCTTCTGAGACCTGGGA

*

1851 1900

VP1-01 (1851) AAAGCATTGTGATTGTGATTCAGTGCTTGATCCATGTCCAGAGTCTTCTG

VP1-02 (1851) AAAGCATTGTGATTGTGATTCAGTGCTTGATCCATGTCCAGAGTCTTCTG

VP1-03 (1851) AAAGCATTGTGATTGTGATTCAGTGCTTGATCCATGTCCAGAGTCTTCTG

1901 1950

VP1-01 (1901) CTTCAGAATCTTCCTCTCTAGGAAAGTCAAGAATGGGTCTCCCCATACCA

VP1-02 (1901) CTTCAGAATCTTCCTCTCTAGGAAAGTCAAGAATGGGTCTCCCCATACCA

VP1-03 (1901) CTTCAGAATCTTCCTCTCTAGGAAAGTCAAGAATGGGTCTCCCCATACCA

1951 2000

VP1-01 (1951) ACATTAGCTTTCATAGTAGAAAATGTATACATGCTTATTTCTAAATCCAG

VP1-02 (1951) ACATTAGCTTTCATAGTAGAAAATGTATACATGCTTATTTCTAAATCCAG

VP1-03 (1951) ACATTAGCTTTCATAGTAGAAAATGTATACATGCTTATTTCTAAATCCAG

2001 2050

VP1-01 (2001) CCTTTCTTTCCACTGCACAATCCTCTCATGAATGGCAGC*AAGGGCGAATT*

VP1-02 (2001) CCTTTCTTTCCACTGCACAATCCTCTCATGAATGGCAGC*AAGGGCGAATT*

VP1-03 (2001) CCTTTCTTTCCACTGCACAATCCTCTCATGAATGGCAGC*AAGGGCGAATT*

2051

VP1-01 (2051) *C*

VP1-02 (2051) *C*

VP1-03 (2051) *C*
